# Blue thermally activated delayed fluorescence emitters incorporating acridan analogues with heavy group 14 elements for high-efficiency doped and non-doped OLEDs†
†Electronic supplementary information (ESI) available. CCDC 1948037–1948040. For ESI and crystallographic data in CIF or other electronic format see DOI: 10.1039/c9sc04492b


**DOI:** 10.1039/c9sc04492b

**Published:** 2019-10-29

**Authors:** Kyohei Matsuo, Takuma Yasuda

**Affiliations:** a INAMORI Frontier Research Center (IFRC) , Kyushu University , 744 Motooka, Nishi-ku , Fukuoka 819-0395 , Japan . Email: yasuda@ifrc.kyushu-u.ac.jp; b Department of Applied Chemistry , Graduate School of Engineering , Kyushu University , 744 Motooka, Nishi-ku , Fukuoka 819-0395 , Japan

## Abstract

Deep-blue thermally activated delayed fluorescence (TADF) emitters are promising alternatives for conventional fluorescence and phosphorescence materials for practical application in organic light-emitting diodes (OLEDs). However, as appropriate bipolar hosts for deep-blue TADF-OLEDs are scarce, the development of efficient deep-blue TADF emitters that are applicable to both doped and non-doped systems is an urgent task. In this study, we developed a new family of blue TADF emitters that demonstrated high photoluminescence (PL) and electroluminescence (EL) quantum efficiencies in both doped and non-doped (neat) systems. Four new donor–acceptor (D–A)-type TADF molecules incorporating phenazasiline, phenazagermine, and tetramethylcarbazole as weak D units and phenothiaborin as a weak A unit were designed and synthesized. By varying the structural rigidity/flexibility as well as the electron-donating ability of the D units, the resulting photophysical and TADF properties of the D–A molecules could be systematically regulated. A comprehensive photophysical investigation revealed that phenazasiline and phenazagermine-based emitters concurrently exhibit blue TADF emissions (464–483 nm), high PL quantum efficiencies (∼100%), extremely fast spin-converting reverse intersystem crossing rates (>10^7^ s^–1^), and suppressed concentration quenching. These fascinating features in conjunction produced high-performance doped and non-doped blue TADF-OLEDs. The doped and non-doped TADF-OLEDs using the phenazasiline-based emitter demonstrated extremely high maximum external EL quantum efficiencies (*η*_ext_) of 27.6% and 20.9%, with CIE chromaticity coordinates of (0.14, 0.26) and (0.14, 0.20), respectively. Further, ultra-low efficiency roll-off behavior for both the doped and non-doped devices was demonstrated by their *η*_ext_ as high as 26.1% and 18.2%, respectively, measured at a practically high luminance of 1000 cd m^–2^.

## Abstract 2

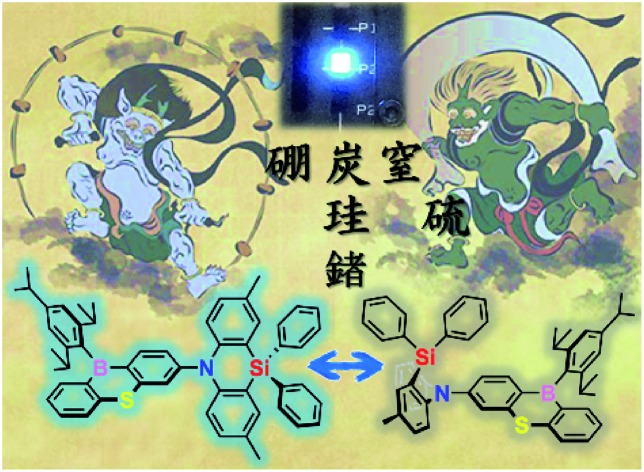
Blue thermally activated delayed fluorescence emitters incorporating phenazasiline and phenazagermine as donor units are developed, and their structural, photophysical, and electroluminescent properties are systematically investigated.

## Introduction

The development of high-performance blue organic light-emitting diodes (OLEDs) is of vital importance for practical applications in full-color flat-panel displays and white lighting sources, and has been attracting growing interest both academically and commercially.^1^ In recent years, considerable research efforts have been devoted to boosting the exciton utilization for electroluminescence (EL) and thereby making a leap forward in the resulting device efficiency. Phosphorescent emitters based on noble metal complexes have become indispensable for this task since 1998,^2^ because of their capability of harvesting both singlet and triplet excitons with almost 100% internal quantum efficiency (*η*_int_) in OLEDs. Among the three primary colors, green and red phosphorescent emitters have already shown high performance in terms of both efficiency and stability, and have been successfully incorporated into commercial OLED products. Recently, highly efficient deep-blue phosphorescent OLEDs with narrow EL emissions have been produced by employing rigid tetradentate Pt(ii) complexes^3^ and *N*-heterocyclic carbene coordinated Ir(iii) complexes.^4^ However, even state-of-the-art deep-blue phosphorescent OLEDs have still lagged behind the green and red counterparts in their operational lifetime and stability, and thus, further development of deep-blue emitters *via* innovative molecular design remains an ongoing challenge.

The replacement of noble metal-containing phosphors with metal-free pure organic fluorophores is a challenging task, but one of high priority, for realizing future low-cost OLED applications. It was not until 2012 that purely organic thermally activated delayed fluorescence (TADF) emitters were first adopted in OLEDs^5^ and demonstrated high *η*_int_ of nearly 100%. Over the last few years, a number of organic TADF materials displaying various emission colors have been developed, and TADF-OLEDs with high external EL quantum efficiencies (*η*_ext_) of up to ∼38%, comparable to those of phosphorescent OLEDs, have been achieved.^6^ However, deep-blue TADF materials capable of exhibiting high *η*_ext_ exceeding 20% (corresponding to *η*_int_ approximating 100%) in OLEDs as well as appropriate color purity (with Commission Internationale de l'Eclairage (CIE) chromaticity coordinates of *x* ≤ 0.15 and *y* ≤ 0.20) are still scarce.^7^

To achieve deep-blue EL, TADF molecules are required to have a wide energy gap between the highest occupied molecular orbital (HOMO) and lowest unoccupied molecular orbital (LUMO), as well as high lowest-excited singlet (S_1_) and triplet (T_1_) energies (≥2.8 eV). Typically, TADF molecules adopt a donor–acceptor (D–A) electronic system, in which the HOMO and LUMO are mainly distributed on the donor and acceptor moieties, respectively, giving rise to intramolecular charge-transfer (ICT) states. Therefore, the combination of a donor having a deeper HOMO level with an acceptor having a shallower LUMO level is prerequisite for the design of deep-blue TADF emitters. While various types of acceptor units, such as triazine,7c,f,j pyrimidine,7*d* diphenylsulfone,7*a* benzophenone,7*g* triarylborane,7h,i and heteraborins,7b,k,l have been used for the design of deep-blue TADF molecules, candidates for relatively weak donors have been limited to carbazole and 9,10-dihydroacridine (acridan) derivatives, which restricts diversity in material design. In this regard, 5,10-dihydrodibenzo[*b*,*e*][1,4]azasiline (phenazasiline), which is a heavy-atom analogue of acridan, has recently been introduced for the design of blue TADF emitters by Kim and co-workers.7c,^8^ As the C–Si bonds, which are longer than C–C bonds, reduce the electron-donating effect arising from hyperconjugation, phenazasiline can function as a weaker donor compared to acridan, and lead to blue-shifted emissions. So far, there are no systematic studies on the structural, electronic, and photophysical properties of TADF emitters based on acridan-analogous donors with heavier group 14 elements.

Another inherent bottleneck for developing efficient and stable deep-blue TADF-OLEDs is the lack of suitable host materials with a high triplet energy (*E*_T_) and balanced charge transport properties. An ideal host for deep-blue TADF emitters requires a high *E*_T_ level surpassing 3.0 eV to facilitate triplet energy transfer from the host to the emitters and prevent back energy transfer. However, it is technically difficult to obtain bipolar high-*E*_T_ host materials owing to the limited availability of suitable building blocks.^9^ This issue can be circumvented by adopting non-doped (neat) emission layers instead of conventional guest–host systems. The non-doped devices require advanced blue TADF emitters with (i) a high photoluminescence (PL) quantum yield (*Φ*_PL_) in the neat film, (ii) short excited-state lifetime to alleviate concentration quenching, and (iii) a favorable bipolar charge transport property. Except for a few recent examples,^10^ most reported blue TADF emitters bearing carbazole-based donors undergo severe aggregation-caused emission quenching (ACQ) and red-shift or broadening of the PL emission caused by intermolecular interactions in the condensed phase. Recent works by our group7b,^11^,^12^ and Wu, Wong, and co-workers^13^ reported non-doped and heavily-doped TADF-OLEDs with acridan-based emitters, which achieved rather high maximum *η*_ext_ values. We also revealed the ACQ mechanism for TADF systems and succeeded in retaining high *Φ*_PL_ in the pure neat films without using any host materials.11*a* Hence, the utilization of acridan-based donors is regarded as a versatile design strategy for efficient non-doped TADF systems; however, achieving blue TADF with a CIE-*y* value (≤0.20) remains a major challenge because of the strong electron-donating characteristics of the acridan-based donors. More recently, aggregation-induced delayed fluorescence (AIDF) has been proposed as a new viable methodology to avoid ACQ and hence produce efficient non-doped OLEDs.^14^ However, thus far, efficient deep-blue AIDF systems that are capable of fulfilling the aforementioned criteria have not been attained.

**Fig. 1:**
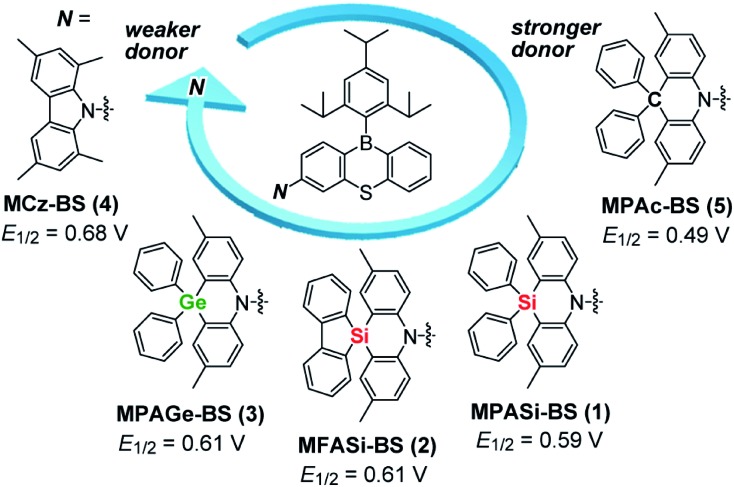
Molecular structures of blue phenothiaborin-based TADF emitters incorporating acridan-analogous donors with different group 14 elements. *E*_1/2_ values represent the half-wave oxidation potentials *vs.* Fc/Fc^+^ determined by cyclic voltammetry and differential pulse voltammetry (ESI†), as a measure of the electron-donating ability.

In this study, we focused on the electronic and structural features of a set of acridan-analogous donors with different group 14 (crystallogen) elements, and systematically designed new TADF emitters **1–4** by combining them with phenothiaborin (BS) as the common acceptor unit (Fig. 1). Having weakened electron-donating abilities, **1–4** indeed showed blue-shifted TADF emissions (by 20–35 nm) in comparison to that of the parent acridan-appended TADF emitter **5**.^12^ In addition, effects of their unique conformational heterogeneity on the photophysical properties and EL characteristics were systematically investigated by changing the bridging group 14 elements for the phenazasiline- and phenazagermine-appended TADF emitters. Of this new family of blue TADF emitters, **1** and **3** were found to exhibit suppressed ACQ behavior and therefore high *Φ*_PL_ values even in their neat films. Owing to this advantageous attribute, non-doped TADF-OLEDs based on **1** and **3** demonstrated remarkable EL performances with high maximum *η*_ext_ of 20.9% and 17.4%, respectively, and blue emission CIE coordinates of (0.14, 0.20), along with suppressed efficiency roll-off. This study provides a new universal guideline for the design of high-performance blue TADF materials applicable to both doped and non-doped blue OLEDs.

## Results and discussion

### Synthesis and characterization

**Scheme 1:**
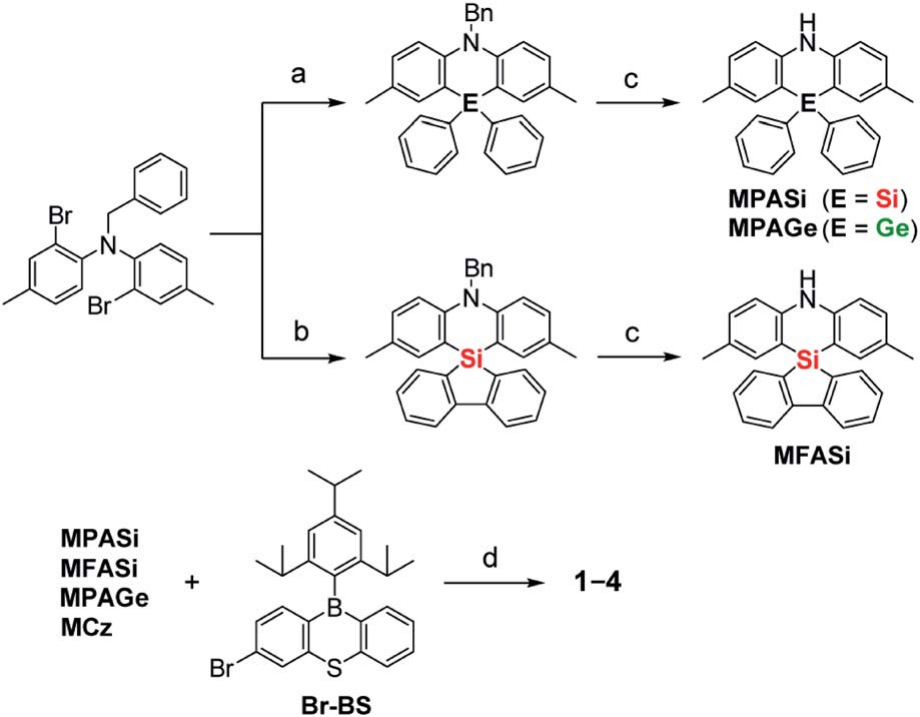
Synthetic schemes for **1–4**. Reagents and conditions: (a) (i) *n*-BuLi, Et_2_O, 0 °C, 1 h, (ii) Ph_2_ECl_2_ (E = Si, Ge), 35 °C, 2 h; (b) (i) *n*-BuLi, Et_2_O, 0 °C, 1 h, (ii) 5,5-dichlorodibenzo[*b*,*d*]silole, 35 °C, 2 h; (c) H_2_, Pd/C, CH_2_Cl_2_/AcOH, RT, 15–24 h; (d) Pd_2_(dba)_3_, *t*-Bu_3_PH·BF_4_, NaO*t*-Bu, toluene, 100 °C, 6–17 h. Bn = benzyl, MCz = 1,3,6,8-tetramethylcarbazole.

Aiming to produce efficient deep-blue TADF emitters, green-emitting MPAc-BS (**5**)^12^ was selected as a prototypical material (Fig. 1) and its donor unit was displaced by less electron-donating analogues such as 2,8-dimethyl-10,10-diphenylphenazasiline (MPASi for **1**), spiro[2,8-dimethylphenazasiline-10,5′-dibenzo[*b*,*d*]silole] (MFASi for **2**), and 2,8-dimethyl-10,10-diphenylphenazagermine (MPAGe for **3**), as well as 1,3,6,8-tetramethylcarbazole (MCz for **4**). The target compounds **1–4** were synthesized through the procedures outlined in Scheme 1. The detailed synthetic procedures and chemical characterization data are given in ESI.† The *N*-benzyl-protected precursors were prepared by dilithiation of *N*,*N*-bis(2-bromo-4-methylphenyl)benzylamine, followed by nucleophilic substitution reactions with dichlorodiphenylsilane, 5,5-dichlorodibenzo[*b*,*d*]silole, or dichlorodiphenylgermane. The catalytic hydrogenation reactions afforded the deprotected donors (MPASi, MFASi, and MPAGe). Finally, **1–4** were obtained *via* Buchwald–Hartwig amination of the mono-brominated phenothiaborin acceptor (Br-BS) with the corresponding donors in good yields (58–89%). Compounds **1–4** showed sufficient tolerance toward air, moisture, and heat, and could be purified by column chromatography and temperature-gradient vacuum sublimation. Thermogravimetric analyses revealed that **1–4** had good thermal stability, with 5% weight-loss temperatures (*T*_d_) of 368, 380, 371, and 343 °C, respectively (ESI†), rendering these materials suitable for the fabrication of OLEDs using the vacuum deposition method.

The variations of the electron-donating ability for the present acridan analogues with different bridging group 14 elements along with MCz were verified by cyclic voltammetry and differential pulse voltammetry (Fig. 1 and ESI†). The half-wave oxidation potentials (*E*_1/2_*vs.* Fc/Fc^+^) increased in the order of **5** (0.49 V) < **1** (0.59 V) < **2** (0.61 V) ≈ **3** (0.61 V) < **4** (0.68 V); this trend reflects the variation of their electron-donating ability. As compared to the case of acridan-appended **5**, the introduction of heavier group 14 elements (*i.e.*, Si for **1** and **2**, and Ge for **3**) gradually elevated *E*_1/2_ to more positive potentials, indicating less electron-donating features of the MPASi, MFASi, and MPAGe donors. As expected, introducing MCz donor made it more difficult for **4** to be electrochemically oxidized, which was indicative of its lower electron-donating ability. These redox processes of **1–4** are essentially reversible and stable, which is a favorable attribute especially for their utilization as non-doped emitters.

### Molecular structures and conformational analyses

**Fig. 2:**
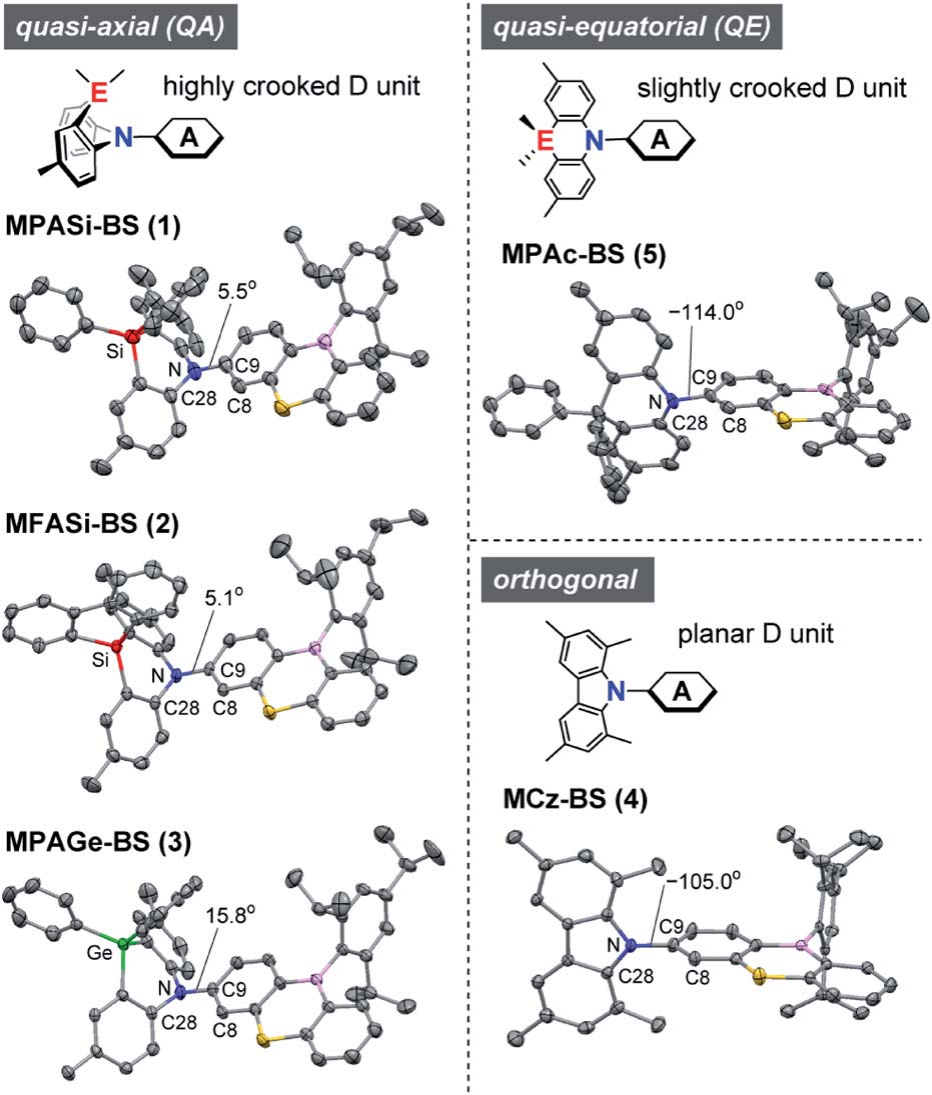
X-ray crystal structures of **1–5** (CCDC ; 1948037–1948040, 1825382†) showing different conformers. Thermal ellipsoids are drawn at 50% probability. Hydrogen atoms, solvent molecules, and disordered isopropyl groups are omitted for clarity. Atom color code: C, gray; B, pink; N, blue; S, yellow; Si, red; Ge, green.

The X-ray crystallographic analyses provided valuable structural information for **1–5** (Fig. 2 and ESI†), which was strongly influenced by the subtle difference in the bridging moiety of the donor units. Unlike the rigid planar MCz donor in **4**, all phenazasiline and phenazagermine donors in **1–3** were largely folded along their central N···Si or N···Ge axis and adopted ‘quasi-axial (QA)’ conformations with small D–A torsion angles (C8–C9–N–C28) of 5.1–15.8° in the crystalline states. The crystal structures of **1–3** were in marked contrast to that of **5**, which preferentially formed a typical ‘quasi-equatorial (QE)’ conformer having a large torsion angle of –114.0°.^15^ Such conformational heterogeneity (*i.e.*, capability to form QA and QE conformers) of this series of D–A molecules can be attributed to the mismatch of the bridging bond lengths in each acridan-analogous donor unit; namely, Ge–C (∼1.94 Å) > Si–C (∼1.86 Å) ≫ C–C (∼1.54 Å) > N–C (∼1.44 Å). Some TADF molecules, most frequently those with a sulfur-containing phenothiazine donor, reportedly show intriguing dual CT emissions arising from two different conformers.^15^,^16^ Despite the similarity in the covalent radii of sulfur and silicon, effects of such conformational heterogeneity of phenazasiline and its derivatives on the optical and electronic properties have never been clarified.

**Fig. 3:**
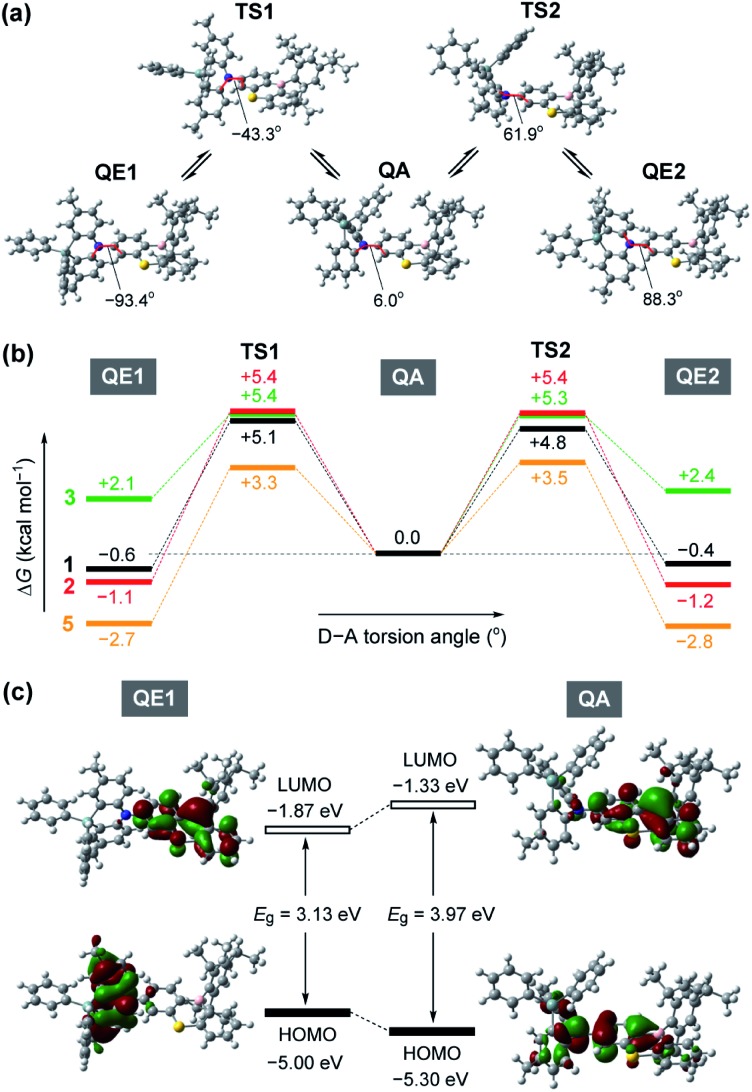
(a) Molecular geometrical changes in **1** along the interconversion between its quasi-equatorial (QE) and quasi-axial (QA) conformers through the transition states (TS). (b) Energy profiles for the interconversion processes of **1–3** and **5** in their ground states at 298 K and 1 atm, calculated at the B3LYP/6-31G(d) level of theory. (c) Spatial distribution of the frontier orbitals (HOMOs and LUMOs) for the ground-state QE and QA conformers of **1**.

To gain further insight into the QA–QE conformational interconversion, we performed computational simulations using density functional theory (DFT), as presented in Fig. 3 (see ESI for details†). For the MPASi-appended **1** as a representative example, three stable conformations with different D–A torsion angles were predicted in the ground (S_0_) state, which corresponded to one QA and two QE conformers (Fig. 3a). The torsion angle of the QA conformer for **1** was as small as 6.0°, consistent with the foregoing crystal structure. In contrast, much larger torsion angles (–93.4° and 88.3°) were estimated for its two QE conformers (QE1 and QE2). In this case, the free energy differences (Δ*G*) between the QA and QE conformers were estimated to be considerably small (<0.6 kcal mol^–1^). The interconversion energy barriers calculated for **1** (4.8–5.7 kcal mol^–1^) appeared to be small enough for allowing free conformational changes in solution at room temperature. Likewise, the geometry optimizations for **2**, **3**, and **5** performed following the same protocol indicated three local minima corresponding to one QA and two QE conformers (Fig. 3b). For **5** with the less crooked MPAc donor, two QE conformers were further energetically stabilized (by 2.7–2.8 kcal mol^–1^) with respect to the corresponding QA conformer. This suggests that the QE conformer with the nearly orthogonal D–A arrangement should have a higher population at equilibrium. Contrary to **1**, **2**, and **5**, the calculated free energies of the two QE conformers for **3** were higher than the QA one (by 2.1–2.4 kcal mol^–1^), implying that the QA conformation was thermodynamically more preferable for the phenazagermine derivative. As the interconversion energy barriers did not change significantly with the variation of the donor units, the population ratios of the QA and QE conformers for **1**, **2**, **3**, and **5** could be estimated using their Δ*G* values by assuming Boltzmann distribution. Consequently, for **1**, **2**, and **5**, the orthogonal QE conformers were expected to have much higher populations (QE = 70%, 87%, and 99%, respectively); in contrast, the QA conformer for **3** predominated over the QE conformer (QA = 98% and QE = 2%) at room temperature. These results unambiguously indicate that a structural modification in the bridging moiety of the donor units has a marked impact on the intrinsic conformational flexibility and heterogeneity.

Fig. 3c depicts the HOMO and LUMO distributions of both QE and QA conformers of **1** in the S_0_ state. Similar to common TADF molecules with an orthogonal D–A arrangement, there was negligible spatial HOMO–LUMO overlap in the QE conformer for **1**, in which the HOMO and LUMO were localized on the phenazasiline donor and phenothiaborin acceptor moieties, respectively, exhibiting evident ICT characteristics. In contrast, the QA conformation of **1** could reduce the D–A distortion and thereby facilitate the conjugation of the lone pair of the nitrogen atom with the adjacent phenothiaborin moiety. Consequently, its HOMO and LUMO were overlapped on the acceptor moiety, giving them a more localized electronic transition character. Furthermore, this QA conformer was found to have a deeper (or more stabilized) calculated HOMO level and also wider HOMO–LUMO energy gap (*E*_g_) compared to the QE conformer. Very similar computational results were obtained for **2** and **3** as well (ESI†), revealing the notably different electronic transition characteristics between the interconvertible QE and QA conformers.

### Photophysical properties in solutions

**Fig. 4:**
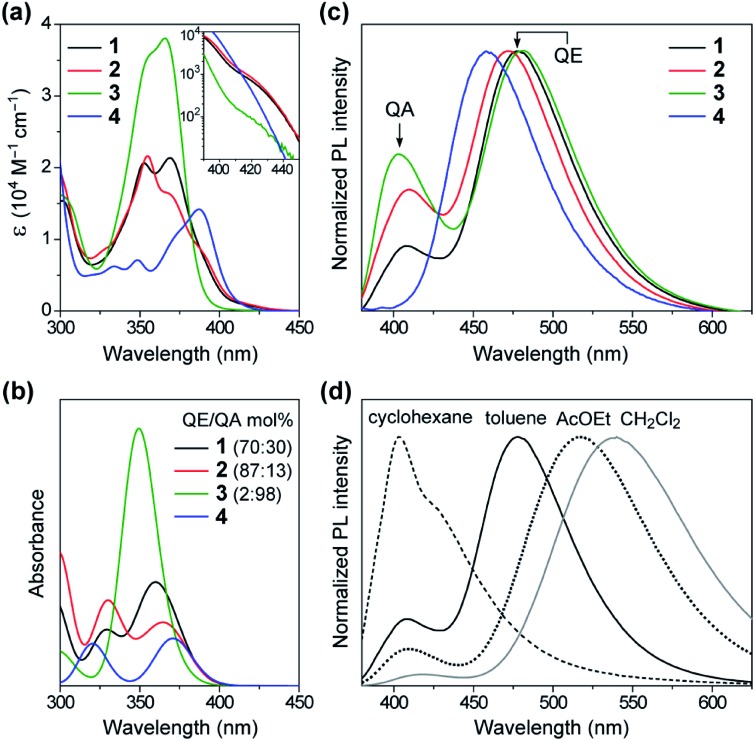
(a) UV-vis absorption spectra of **1–4** in toluene (10^–5^ M). The inset shows a magnified view of the lower-energy ICT absorption bands. (b) Theoretical absorption spectra of **1–4** with different populations of QE/QA conformers simulated using TD-DFT (LC-ωPBE/6-31+G(d)). (c) Steady-state PL spectra of **1–4** in the deoxygenated toluene solutions. (d) Solvatochromic PL responses of **1** in different solvents upon excitation at 350 nm.

Fig. 4a shows a comparison of the UV-vis absorption spectra of **1–4** in toluene solutions. The phenazasiline-appended **1** and **2** exhibited very weak absorption bands in a lower-energy region of 390–430 nm, in addition to major intense absorptions ranging from 330 to 390 nm. Meanwhile, the phenazagermine-appended **3** exhibited much stronger absorption bands centered at 366 nm with a molar absorption coefficient (*ε*) as high as 3.8 × 10^4^ M^–1^ cm^–1^. To further understand the origin of their different absorption behavior, the absorption spectra of **1–4** were theoretically simulated by taking the population ratios of QA/QE conformers into account, using time-dependent DFT (TD-DFT) with the long-range corrected functional (LC-ωPBE)^17^ and the 6-31+G(d) basis set (Fig. 4b and ESI†). It was found that the electronic excitations of the QE conformers occurred at lower energies than those of the QA conformers in the calculations, even though the oscillator strength of the former is close to zero. On this basis, the weak absorption bands observed with **1** and **2** in the range of 390–430 nm were assigned to the ICT transitions of their QE conformers. Further, the strong absorption peak at 366 nm in **3** was mainly attributable to the π–π* transition of its predominant QA conformer having a larger oscillator strength. The overall spectral profiles for **1–4** could be theoretically reproduced, thus testifying that the differences in their transition energies and strengths primarily originated from the different population ratios of the QA/QE conformers in the S_0_ states.

The effect of this conformational heterogeneity was also observed in the PL spectra in dilute solutions (Fig. 4c). The MCz-appended **4** exhibited a typical single emission with the peak (*λ*_PL_) at 458 nm and *Φ*_PL_ of 22%, which originated from the twisted ICT state. In contrast, **1–3** clearly displayed dual emissions consisting of a main peak at 472–482 nm (blue region) and a shoulder peak at 403–410 nm (violet region) in deoxygenated toluene solutions (overall *Φ*_PL_ = 46%, 27%, 42%, respectively). The corresponding excitation spectra of **1–3** monitored at these two emission bands showed prominent differences (ESI†), supporting the notion that the dual emissions arose from different conformers. To verify the ICT character of the dual emissions, we compared the PL spectra of **1–3** in four solvents of different polarities (Fig. 4d and ESI†). In non-polar cyclohexane, the higher-energy emission centered at ∼400 nm was dominant. Upon increasing the solvent polarity from cyclohexane (*E*_T_(30) = 30.9)^18^ through toluene (33.9) to ethyl acetate (38.1) and dichloromethane (40.7), the relative intensity of the higher-energy emission was sharply reduced, and at the same time, the lower-energy broad emission emerged and became predominant. Furthermore, the lower-energy emission showed significant bathochromic shift (*i.e.*, positive solvatochromism) with an increase in the solvent polarity, indicating obvious ICT nature. On the other hand, the higher-energy emission underwent less solvatochromic effect, as a consequence of its locally excited (LE) or mixed LE and ICT character. This solvent-dependent dual PL behavior in **1–3** thus indicated that the higher- and lower-energy emissions primarily originated from the QA and QE conformers having different electronic characteristics, respectively.

**Fig. 5:**
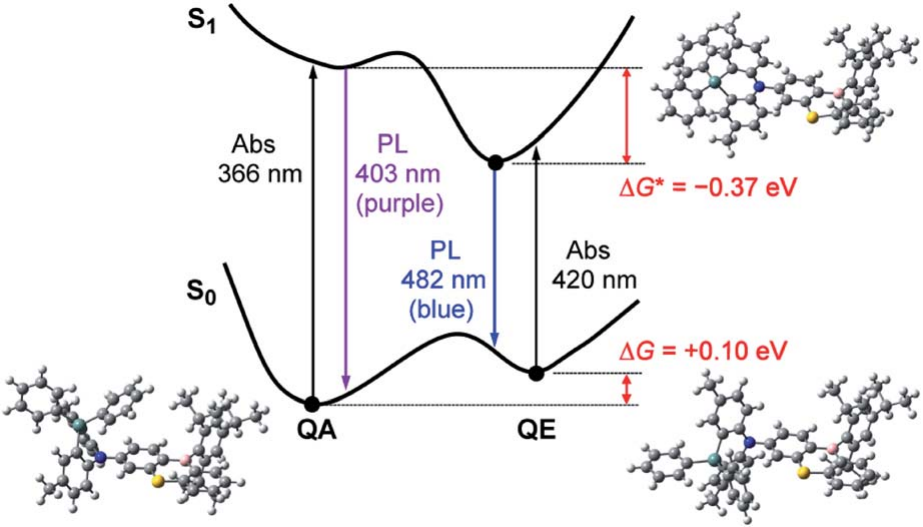
Schematic energy level diagram for **3** exhibiting the mechanism of excited-state interconversion and dual PL emissions in toluene solution. The optimized structures of the QA and QE conformations in the S_0_ state and the orthogonal conformation in the S_1_ state calculated at the B3LYP/6-31G(d) level are presented.

However, one question now arises for the phenazagermine-appended **3**: why did the QE emission become predominant over the QA emission in spite of the negligible population of its QE conformer in the S_0_ state? This may be caused by a change in the thermodynamic equilibrium between the QA and QE conformers of **3** in the S_1_ state. According to the Förster cycle,^19^ the energy differences (ΔG*) between the QA and QE conformers in the S_1_ state can be estimated by the following equation:

1 ΔG* = Δ*G* – (Δ*E*_QA_ – Δ*E*_QE_)

where Δ*E*_QA_ and Δ*E*_QE_ are the excitation energies of the QA and QE conformers, respectively. The Δ*E*_QA_ and Δ*E*_QE_ values of **3** were estimated as 3.19 and 2.76 eV, respectively, from the average of the corresponding absorption and emission maxima. Given the Δ*G* of +0.10 eV (Fig. 3b), ΔG* of **3** was calculated to be –0.37 eV. As schematically shown in Fig. 5, the QE conformer is energetically more stable than the QA conformer in the S_1_ state, likely owing to its prominent ICT character. Therefore, the QA conformer readily undergoes excited-state structural relaxation (or photo-induced isomerization) and transforms into the QE conformer beyond a small energy barrier to emit the lower-energy blue PL.16*g* Indeed, the optimized S_1_ geometries of **1–3** were calculated to have orthogonal D–A structures with the torsion angle of almost 90° (Fig. 5 and ESI†), similar to the corresponding ground-state QE conformers. This computational result supports the experimental observation of the predominant blue emissions from the QE conformers in the solution PL spectra of **1–3**.

### Photophysical properties and exciton dynamics in thin films

**Fig. 6:**
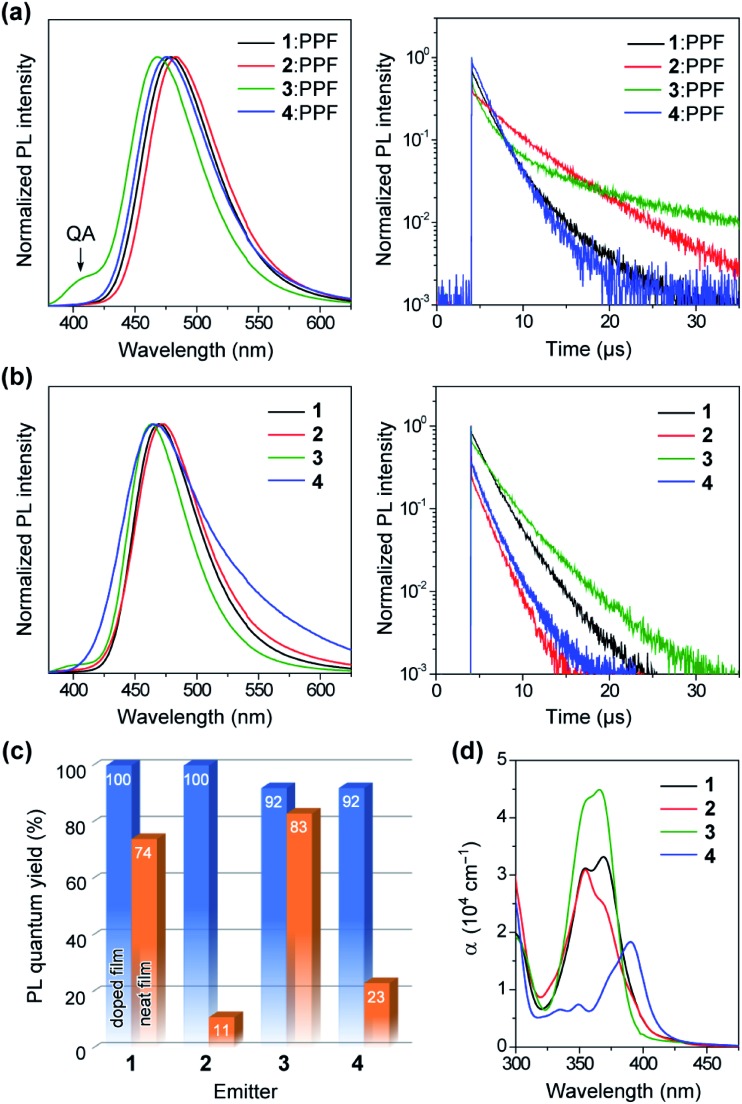
(a and b) Steady-state PL spectra (left) and transient PL decay profiles (right) of **1–4** in (a) 50 wt%-emitter:PPF doped films and (b) pure neat films measured at 300 K under N_2_. (c) Comparison of *Φ*_PL_ values measured with **1–4** in the doped and neat films. (d) UV-vis absorption spectra of the neat films of **1–4**.

To unveil their potential as blue TADF emitters prior to device fabrication, the photophysical properties of **1–4** were investigated in both doped and neat films, as presented in Fig. 6. For doped films, bis(diphenylphosphoryl)dibenzo[*b*,*d*]furan (PPF, *E*_T_ = 3.1 eV) was employed as a wide-gap high-T_1_ host to effectively confine the S_1_ and T_1_ excitons in the TADF emitters. The detailed photophysical data are summarized in Table 1. All doped films of **1–4** displayed intense blue PL emissions (*λ*_PL_ = 468–483 nm) with notably high absolute *Φ*_PL_ values of 92–100% under N_2_ atmosphere (Fig. 6a and c). Interestingly, unlike dilute solutions, the emission from the QA conformers completely disappeared in the doped films of **1** and **2**. Given that large structural relaxation cannot occur easily in the solid state, the intensified QE emissions likely originated for another reason. Because of the spectral overlap between the QA emission and QE absorption, the excited energy of the QA conformer could be transferred to the QE conformer by Förster resonance energy transfer (FRET), dissipating the emission from the QA conformer in these doped films. However, only the doped film of **3** exhibited a clear QA emission at ∼400 nm as a shoulder, which was ascribed to an incomplete FRET due to a tiny proportion of the QE conformer in its initial S_0_ equilibrium. The absorption spectra of the neat films of **1–3** (Fig. 6d) were nearly the same as those measured in toluene solution, which suggests that the neat films were composed of mixtures of their QA and QE conformers having population ratios equivalent to those in the solution. As listed in Table 1, the Δ*E*_ST_ values of **1–4** were estimated as small as 0.08, 0.06, 0.11, and 0.11 eV, respectively, from the energy differences between the onsets of the fluorescence and low-temperature phosphorescence spectra of the doped films (ESI†).

**Table 1:** Photophysical data of **1–4** in doped and neat films

Emitter^*a*^		*λ*_PL_ ^*b*^ [nm]	*E*_FWHM_ ^*c*^ [eV]	*λ*_FWHM_ ^*c*^ [nm]	*Φ*_PL_ ^*d*^ [%]	*Φ*_p_ ^*e*^ [%]	*Φ*_d_ ^*e*^ [%]	*τ*_p_ ^*f*^ [ns]	*τ*_d_ ^*f*^ [μs]	*k*_r_ ^*g*^ [10^6^ s^–1^]	*k*_ISC_ ^*h*^ [10^8^ s^–1^]	*k*_RISC_ ^*i*^ [10^7^ s^–1^]	Δ*E*_ST_^*j*^ [eV]
**1**	Dope	479	0.36	69	100	1.1	98.9	2.9	2.7	3.9	3.5	3.3	0.08
	Neat	469	0.34	62	74	0.6	73.4	0.95	2.3	6.1	10	5.4	
**2**	Dope	483	0.35	68	100	1.4	98.6	3.4	5.3	4.1	2.9	1.3	0.06
	Neat	473	0.35	68	11	0.8	10.2	1.3	1.7	6.4	7.8	0.74	
**3**	Dope	468	0.37	67	92	0.5	91.5	1.7	10.4	3.2	5.9	1.6	0.11
	Neat	464	0.32	57	83	0.7	82.3	0.95	3.2	7.0	10	3.9	
**4**	Dope	476	0.37	70	92	0.8	91.2	1.7	1.9	4.7	5.9	5.9	0.11
	Neat	465	0.46	85	23	0.4	22.6	0.54	1.8	6.6	18	3.6	

^*a*^Measured as a 50 wt% doped thin film in a PPF host matrix (Dope) or pure neat film (Neat) at 300 K under N_2_.

^*b*^PL emission maximum.

^*c*^Full width at half-maximum of the PL spectrum given in energy or wavelength.

^*d*^Absolute PL quantum yield evaluated using an integrating sphere from overall QA and QE emissions.

^*e*^Fractional quantum yields for prompt fluorescence (*Φ*_p_) and delayed fluorescence (*Φ*_d_): *Φ*_p_ + *Φ*_d_ = *Φ*_PL_.

^*f*^Emission lifetimes for prompt fluorescence (*τ*_p_) and delayed fluorescence (*τ*_p_).

^*g*^Rate constant of fluorescence radiative decay (S_1_ → S_0_): *k*_r_ = *Φ*_p_/*τ*_p_.

^*h*^Rate constant of ISC (S_1_ → T_1_): *k*_ISC_ = (1 – *Φ*_p_)/*τ*_p_.

^*i*^Rate constant of RISC (T_1_ → S_1_): *k*_RISC_ = *Φ*_d_/(*k*_ISC_*τ*_p_*τ*_d_*Φ*_p_).

^*j*^Singlet–triplet energy splitting determined from the lowest excited singlet (*E*_S_) and triplet (*E*_T_) energies by Δ*E*_ST_ = *E*_S_ – *E*_T_ (see ESI).

Interestingly, the PL spectra of **1–3** were blue-shifted and became narrower in the neat films (Fig. 6b and Table 1), as compared to those of the doped films. This behavior can be explained by the difference in the polarity of the surrounding solid host media. Compared to the highly polar PPF host, the less polar nature of **1–3** as host media implied a lower degree of stabilization for the polar ICT excited states, thus leading to hypsochromic shifts of PL emissions in the neat films. Moreover, owing to their terminal bulky substituents, **1** and **3** were capable of suppressing ACQ and thereby exhibited considerably high *Φ*_PL_ of 74% and 83%, respectively, even in the aggregated neat films (Fig. 6c). This feature is favorable for the fabrication of non-doped blue OLEDs (as will be discussed later). In contrast, the neat film of **4** bearing the planar MCz donor gave a broadened PL spectrum and a much lower *Φ*_PL_, similar to the cases of conventional TADF emitters, presumably because of intermolecular electron-exchange interactions between the neighboring MCz moieties in the aggregated state.11*a*

To verify the TADF nature, the transient PL characteristics of **1–4** were examined in both doped and neat films (Fig. 6a and b, right). All transient PL decay curves could be fitted using a double-exponential model, giving prompt fluorescence lifetimes in the nanosecond regime (*τ*_p_ = 0.5–3.4 ns) and delayed fluorescence lifetimes in the microsecond regime (*τ*_d_ = 1.7–10.4 μs). It is worth noting that each decay curve of the doped films contained a trace portion of prompt fluorescence with a very small fractional quantum yield (*Φ*_p_ = 0.5–1.5%) while most of the PL originated from delayed fluorescence (*Φ*_d_ = 91–99%). This ideal TADF behavior can be attributed to their capability of fast intersystem crossing (ISC) and reverse ISC (RISC). Indeed, extremely large ISC and RISC rate constants (*k*_ISC_ and *k*_RISC_) exceeding 10^8^ and 10^7^ s^–1^ were evaluated for **1–4**, as listed in Table 1. These *k*_ISC_ and *k*_RISC_ values were approximately one and two orders of magnitude greater, respectively, than the corresponding fluorescence radiative decay rate constant (*k*_r_ ∼ 10^6^–10^7^ s^–1^). Therefore, the rate-limiting step in the TADF processes of **1–4** is now the fluorescence radiative decay (S_1_ → S_0_) rather than RISC (T_1_ → S_1_), which is completely opposite to conventional TADF materials that have much lower *k*_RISC_ (<10^6^ s^–1^). To the best of our knowledge, the *k*_RISC_ values obtained for **1–4** (>10^7^ s^–1^) are the highest among all TADF emitters ever reported, irrespective of the emission colors. This result is closely associated with the fact that the *Φ*_d_ dominated the overall *Φ*_PL_ values, enabling nearly 100% ISC efficiencies (*Φ*_ISC_ = 1 – *Φ*_p_) and 92–100% RISC efficiencies (*Φ*_RISC_ = *Φ*_d_/*Φ*_ISC_) for **1–4**.^20^

**Fig. 7:**
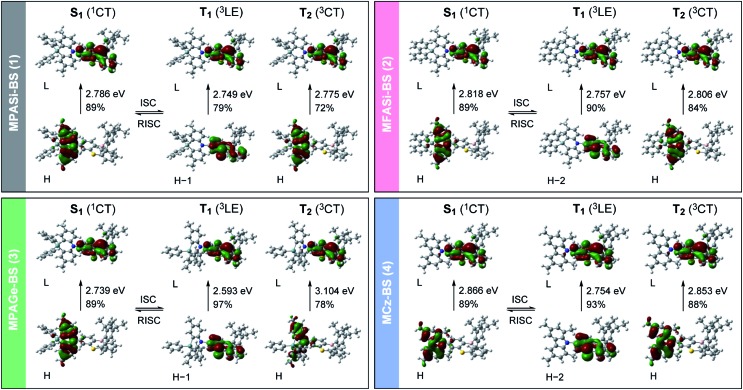
Electronic transition characteristics and associated frontier orbital distributions for the excited S_1_, T_1_, and T_2_ states of **1–4** calculated using TD-DFT (LC-ωPBE/6-31+G(d)) at the optimized S_1_ or T_1_ geometries. The values on each arrow represent the adiabatic excitation energy and contribution weight of the depicted orbitals (H = HOMO; L = LUMO).

We further investigated the excited-state electronic properties of **1–4** using TD-DFT (LC-ωPBE/6-31+G(d)) calculations. As can be seen from Fig. 7, the optimized S_1_ states of **1–4** (with the emissive QE conformation) were dominated by the HOMO → LUMO ICT transitions (^1^CT). In contrast, their optimized lowest T_1_ states were mainly associated with the HOMO–1 (or HOMO–2) → LUMO transitions (^3^LE), corresponding to the π–π* transition of the phenothiaborin acceptor moiety. Indeed, the measured *E*_T_ of the phenothiaborin precursor (Br-BS, 2.78 eV) was nearly the same as those of **1–4** (*E*_T_ = 2.76–2.81 eV, ESI†), supporting the implication that the π–π* transition of the phenothiaborin moiety was from their most relevant T_1_ states. The adiabatic Δ*E*_ST_ values for **1–4** were calculated to be 37, 61, 146, and 112 meV, respectively, which are in satisfactory agreement with the experimental Δ*E*_ST_ values. It was also found that the upper-lying T_2_ states, which are represented by triplet ICT (^3^CT), lay at slightly higher energies than the corresponding T_1_ states. One can anticipate that RISC is intrinsically allowed between the ^3^LE and ^1^CT states with effective spin–orbit coupling (SOC), CT states with effective spin–orbit coupling (SOC), 〈^1^CT|*H*_SOC_|^3^LELE〉, because of the vanishing of the SOC between , because of the vanishing of the SOC between ^3^CT and ^1^CT states having the same spatial orbital occupation (*i.e.*, , 〈^1^CT|*H*_SOC_|^3^CTCT〉 ≈ 0). ≈ 0).^12^,^21^ The predominant ^3^LE component (79–97%) in the lowest T_1_ states of **1–4** is thus beneficial to the spin-converting RISC processes, in addition to having small Δ*E*_ST_ values.

### OLED device performance

To evaluate the EL performances of **1–4** as blue TADF emitters, we first fabricated doped OLEDs (devices A–D) employing 50 wt%-emitter:PPF doped films as the emission layer (EML). The fabricated device structure was indium tin oxide (ITO, 50 nm)/HAT-CN (10 nm)/TAPC (50 nm)/CCP (10 nm)/EML (20 nm)/PPF (10 nm)/B3PyPB (30 nm)/Liq (1 nm)/Al (100 nm), where 2,3,6,7,10,11-hexacyano-1,4,5,8,9,12-hexaazatriphenylene (HAT-CN), 1,1-bis[4-[*N*,*N*-di(*p*-tolyl)amino]phenyl]cyclohexane (TAPC), 9-phenyl-3,9′-bicarbazole (CCP), 1,3-bis[3,5-di(pyridin-3-yl)phenyl]benzene (B3PyPB), and 8-hydroxyquinolinolato lithium (Liq) layers play the roles of hole injection, hole transport, exciton and electron block, electron transport, and electron injection, respectively. A detailed energy level diagram of the devices is provided in ESI.† As the HOMO and LUMO energy levels of emitters **1–4** (*E*_HOMO_ = –5.7 to –5.9 eV and *E*_LUMO_ = –2.7 to –3.0 eV) are shallower and deeper, respectively, than those of the PPF host (–6.7 and –2.7 eV), electrically injected holes and electrons can be directly trapped on the guest emitter molecules to effectively generate excitons with in the EML.

**Fig. 8:**
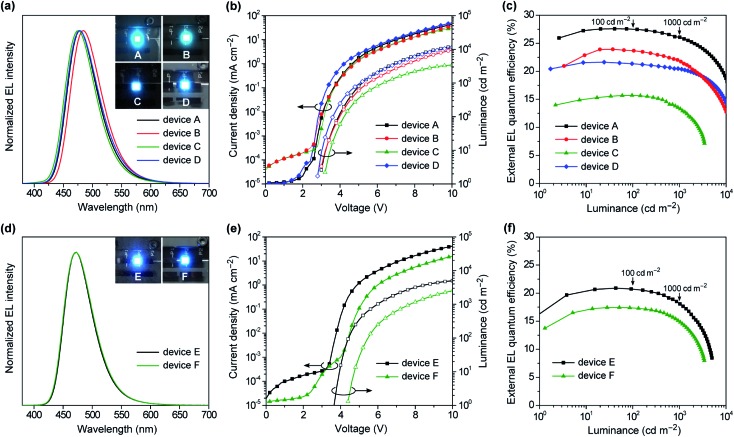
EL characteristics for (a–c) doped TADF-OLEDs (devices A–D) based on 50 wt% **1–4**:PPF and (d–f) non-doped TADF-OLEDs (devices E and F) based on **1** and **3**: (a and d) EL spectra measured at 10 mA cm^–2^ and photos of blue EL emissions, (b and e) *J*–*V*–*L* characteristics, and (c and f) *η*_ext_*versus L* plots.

Fig. 8a–c shows the EL spectra, current density–voltage–luminance (*J*–*V*–*L*) characteristics, and *η*_ext_*versus L* plots for the doped devices A–D, while Table 2 compiles the key EL parameters. Devices A–D displayed blue to sky-blue EL with the emission peaks (*λ*_EL_) ranging from 476 to 484 nm, similar to the corresponding PL spectra. The turn-on voltages (*V*_on_) of these devices were as low as 2.8–3.2 V, which were comparable to the photo-excited S_1_ energies of **1–4** (*E*_S_ = 2.8–2.9 V), suggesting a direct charge recombination and exciton generation mechanism. Comparing the performances of the TADF emitters **1–4** among devices A–D, the maximum *η*_ext_ values were in the order of **1** (27.6%) > **2** (23.9%) > **4** (21.6%) > **3** (15.7%). Particularly, device A using **1** achieved the best EL performance with a maximum *η*_ext_ of 27.6%, current efficiency (*η*_c_) of 48.7 cd A^–1^, power efficiency (*η*_p_) of 48.7 lm W^–1^, and CIE coordinates of (0.14, 0.26). Furthermore, device A demonstrated ultra-low efficiency roll-off; the *η*_ext_ values remained as high as 27.5% at 100 cd m^–1^ (for display), 26.1% at 1000 cd m^–1^ (for lighting), and 18.7% even at 10000 cd m^–1^, corresponding to approximately 0.3%, 5%, and 32% roll-offs, respectively, relative to the maximum *η*_ext_ value. This suppressed efficiency roll-off in device A is even smaller than those of state-of-the-art blue TADF-OLEDs.^7^ In spite of the same *Φ*_PL_ values for the doped films of **3** and **4**, device C using **3** exhibited much lower *η*_ext_ (≤15.5%) over the entire luminance range, which was attributed to the low population of its emissive QE conformer causing a reduced exciton generation probability, even in the heavily-doped EML. In device C, the excitons are formed mainly on the major QA conformer of **3**, and then excited energy transfer to the emissive QE conformer takes place. As the population of the QE conformer is significantly low, the generated excitons are lost to some extent *via* incomplete energy transfer, resulting in lower EL efficiencies.15*b* In addition, the different length dependence of the energy transfer processes for singlet and triplet excitons seems to be relevant to the discrepancy of the trend between the PL and EL efficiencies.^22^ Since only the singlet excitons are initially generated upon photoexcitation, effective energy transfer from the QA to QE conformers takes place *via* long-range Förster mechanism and hence, efficient TADF emission from the QE conformer can be achieved in the PL process with a high *Φ*_PL_ value. In contrast, under electrical excitation in OLEDs, additional short-range Dexter energy transfer, which is much more sensitive to the interchromophore distance, should cause incomplete energy transfer to the emissive QE conformer and lowering the EL efficiency. This can be supported by the fact that the non-doped OLED (device F) with a higher content of the QE conformer within the EML exhibited a higher EL efficiency than the corresponding doped OLED (device C).

**Table 2:** EL performances of the TADF-OLEDs based on **1–4**

Device	EML^*a*^	*λ*_EL_ ^*b*^ [nm]	*E*_FWHM_ ^*c*^ [eV]	*λ*_FWHM_ ^*c*^ [nm]	CIE^*d*^ [*x*, *y*]	*V*_on_ ^*e*^ [V]	*η*_ext,max_ ^*f*^ [%]	*η*_ext,100/1000/10000_ ^*g*^ [%]	*η*_c_ ^*h*^ [cd A^–1^]	*η*_p_ ^*i*^ [lm W^–1^]
A	**1**:PPF	478	0.33	61	(0.14, 0.26)	3.0	27.6	27.5/26.1/18.7	48.7	48.7
B	**2**:PPF	484	0.32	62	(0.14, 0.32)	3.0	23.9	23.7/21.9/12.9	47.8	45.0
C	**3**:PPF	476	0.33	62	(0.14, 0.22)	3.2	15.7	15.5/13.2/—	24.8	22.5
D	**4**:PPF	478	0.34	64	(0.14, 0.26)	2.8	21.6	21.3/20.4/14.4	39.0	41.9
E	**1**	473	0.31	57	(0.14, 0.20)	3.6	20.9	20.7/18.2/—	30.1	23.8
F	**3**	473	0.32	59	(0.14, 0.20)	4.4	17.4	17.4/14.9/—	25.7	16.9

^*a*^Emission layer consisting of a codeposited 50 wt%-emitter:PPF doped film (for devices A–D) or a non-doped neat film (for devices E and F).

^*b*^EL emission maximum at 10 mA cm^–2^.

^*c*^Full width at half-maximum of the EL spectrum given in energy (eV) or wavelength (nm).

^*d*^CIE chromaticity coordinates.

^*e*^Turn-on voltage at a luminance above 1 cd m^–2^.

^*f*^Maximum external EL quantum efficiency.

^*g*^External EL quantum efficiency at the luminance of 100, 1000, and 10000 cd m^–2^.

^*h*^Maximum current efficiency.

^*i*^Maximum power efficiency.

The specifically low ACQ characteristics of **1** and **3** in the neat films prompted us to further evaluate the EL performance in the host-free non-doped devices. To this end, we fabricated the non-doped TADF-OLEDs (devices E and F) by adopting the neat films of **1** and **3** instead of the doped films as the EML, with the same device configuration. Devices E and F exhibited slightly blue-shifted *λ*_EL_ of 473 nm with narrower bandwidths (*E*_FWHM_ = 0.31–0.32 eV and *λ*_FWHM_ = 57–59 nm, Fig. 8d) compared to the corresponding doped devices (A and C). Consequently, both devices E and F achieved blue CIE coordinates of (0.14, 0.20). Remarkably, *η*_ext_ of the non-doped devices E and F reached 20.9% and 17.4%, respectively (Fig. 8e and f and Table 2). Moreover, the efficiency roll-off of device E was rather low, retaining high *η*_ext_ values of 20.7% and 18.2% even when driven at practically high luminances of 100 and 1000 cd m^–1^, respectively, which are among the best performing values for the reported non-doped blue TADF-OLEDs.^10^–^12^ It is also noteworthy that device F using **3** exhibited superior EL performance to the corresponding doped device C. This result may be explained by the increased emissive QE conformers within the non-doped EML.

## Conclusions

In this study, we systematically designed and synthesized new blue TADF emitters featuring acridan-analogous donors containing silicon and germanium bridging atoms. Incorporating larger silicon and germanium atoms enhanced the structural flexibility of the donor units, leading to conformational heterogeneity as well as dual fluorescence capability of the resulting TADF emitters. While all these TADF molecules adopted dual conformations in both solution and thin film states, efficient excited energy transfer from the QA to QE conformers took place and the latter displayed obvious TADF characteristics. As phenazasiline and phenazagermine acted as weaker electron donors than the commonly used acridan unit, bluer TADF was attained. Additionally, the direct electronic coupling between the close-lying local triplet (^3^LE) and charge-transfer singlet (^1^CT) excited states *via* SOC greatly enhanced the RISC rates (*k*_RISC_ > 10^7^ s^–1^) and thereby TADF efficiencies of the emitters. It was also found that blue TADF emitters **1** and **3** with the diphenyl-substituted phenazasiline and phenazagermine donors demonstrated surprisingly small concentration quenching behavior and were applicable to both doped and non-doped systems as efficient bifunctional emitters. The doped and non-doped blue TADF-OLEDs fabricated using **1** demonstrated excellent EL performances, with maximum *η*_ext_ as high as 27.6% and 20.9%, respectively, and suppressed efficiency roll-off at a practically high luminance. We believe that this systematic study can offer a general guideline toward precise control of the structures and photophysical and TADF properties. By utilizing the design concept demonstrated here and improving the intrinsic stability and device architectures, outstanding deep-blue TADF-OLEDs can be developed.

## Conflicts of interest

There are no conflicts to declare.

## Supplementary Material

Supplementary information

Crystal structure data
