# Supplementary material for: Blue thermally activated delayed fluorescence emitters incorporating acridan analogues with heavy group 14 elements for high-efficiency doped and non-doped OLEDs
Source: Chem Sci. 2019 Oct 29;10(46):10687–97. doi: 10.1039/c9sc04492b (PMC7069243; doi:10.1039/c9sc04492b)
Supplement: Supplementary file 1 [file SC-010-C9SC04492B-s001.pdf]

Electronic Supplementary Material (ESI) for Chemical Science.
This journal is © The Royal Society of Chemistry 2019

Electronic Supplementary Information (ESI)

Blue thermally activated delayed fluorescence emitters incorporating acridan analogues with heavy group 14 elements for high-efficiency doped and non-doped OLEDs

Kyohei Matsuo*[ac] and Takuma Yasuda*[ab]

[a] INAMORI Frontier Research Center (IFRC), Kyushu University,
744 Motooka, Nishi-ku, Fukuoka 819-0395, Japan

[b] Department of Applied Chemistry, Graduate School of Engineering,
Kyushu University, 744 Motooka, Nishi-ku, Fukuoka 819-0395, Japan

[c] Current address: Division of Materials Science,
Nara Institute of Science and Technology (NAIST), 8916-5 Takayama-cho, Ikoma, 630-0192, Japan

[*] E-mail: kmatsuo@ms.naist.jp; yasuda@ifrc.kyushu-u.ac.jp

Table of Contents:

1. Materials, Synthesis, and Characterization S2–S12
2. X-ray Crystallographic Analysis S13–S14
3. Electrochemical Analysis S15
4. Photophysical Properties S16–S21
5. Computational Methods S22–S25
6. OLED Fabrication and Evaluations S26–S27
7. References S27

1. Materials, Synthesis, and Characterization

All reagents and anhydrous solvents were purchased from Sigma-Aldrich, Tokyo Chemical Industry (TCI), or FUJIFILM Wako Pure Chemical Corp., and were used without further purification unless otherwise noted. *N*,*N*-Bis(2-bromo-4-methylphenyl)benzylamine[1] and 3-bromo-10-(2,4,6-triisopropylphenyl)-dibenzo[*b*,*e*][1,4]thiaborin[2] were prepared according to the literature procedures. 2,3,6,7,10,11-Hexacyano-1,4,5,8,9,12-hexaazatriphenylene (HAT-CN) was donated by the Nippon Soda Co., Ltd. and purified by vacuum sublimation before use. Other OLED materials were purchased from E-Ray Optoelectronic Technology Co., Ltd. and were used without further purification. Final products **1–4** were purified by temperature-gradient vacuum sublimation to provide highly pure materials for photophysical measurements and device fabrications. ^{1}H and ^{13}C NMR spectra were recorded on a Bruker Avance III 400 spectrometer (400 MHz, for ^{1}H and 100 MHz for ^{13}C, respectively). Chemical shifts of ^{1}H and ^{13}C NMR signals were quoted to tetramethylsilane (δ = 0.00 ppm) and $CDCl_3$ (δ = 77.00 ppm), respectively, as internal standards. Matrix-assisted laser desorption ionization time-of-flight (MALDI-TOF) mass spectra were collected on a Bruker Daltonics Autoflex III spectrometer using dithranol as matrices. Elemental analyses were carried out with a Yanaco MT-5 analyzer. Thermogravimetric analyses (TGA) were performed using a Hitachi High-Tech Science TG/DTA7300 analyzer at a heating rate of 10 °C min^{-1} under N_2 and 5% weight-loss temperatures (T_d) of **1–4** were determined.

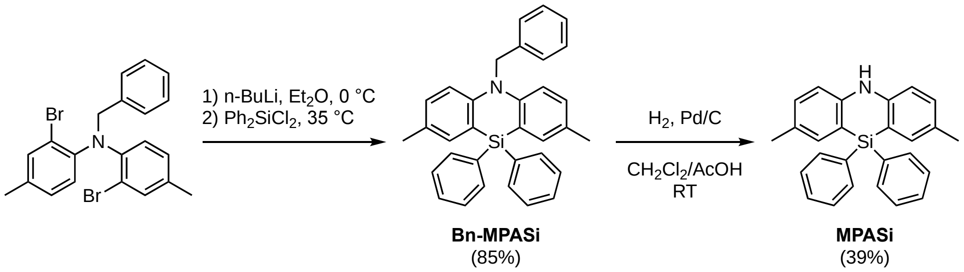

***N*-Benzyl-2,8-dimethyl-10,10-diphenyl-5,10-dihydrodibenzo[*b*,*e*][1,4]azasiline (Bn-MPASi):** To a stirred solution of *N*,*N*-bis(2-bromo-4-methylphenyl)benzylamine (2.67 g, 6.00 mmol) in anhydrous diethyl ether (30 mL) was added dropwise *n*-buthyllithium in hexane (1.51 M, 8.4 mL, 12.7 mmol) at 0 °C under N_2. The mixture was stirred at 0 °C for 1 h, and then, dichrorodiphenylsilane (1.4 mL, 6.75 mmol) was added dropwise. The reaction mixture was stirred at 35 °C for 2 h and then cooled to room temperature. After the addition of water, the product was extracted with AcOEt. The combined organic layers were washed with brine and dried over anhydrous Na_2SO_4. After filtration and evaporation, the crude product was purified by column chromatography on silica gel (eluent: hexane/CH_2Cl_2 = 4:1, v/v). After removal of

the solvents by evaporation, the resulting solid was washed with MeOH to afford **Bn-MPASi** as a white solid (yield = 2.37 g, 85%). ^{1}H NMR (400 MHz, $CDCl_3$): *δ* 7.58–7.56 (m, 4H), 7.44–7.34 (m, 6H), 7.32–7.27 (m, 5H), 7.20 (d, *J* = 6.8 Hz, 2H), 7.06 (dd, *J* = 8.8, 1.6 Hz, 2H), 6.86 (d, *J* = 8.4 Hz, 2H), 5.17 (s, 2H), 2.23 (s, 6H). MS (MALDI-TOF): *m*/*z* calcd 467.21 $[M]^+$; found 467.30.

2,8-Dimethyl-10,10-diphenyl-5,10-dihydrodibenzo[*b,e*][1,4]azasiline (MPASi): A suspension of **Bn-MPASi** (1.87 g, 4.00 mmol) and 5% Pd/C (427 mg) in a mixed solvent of CH_2Cl_2 (10 mL) and AcOH (10 mL) was vigorously stirred under H_2 atmosphere at room temperature for 24 h. After the reaction mixture was filtered through a Celite® pad, the filtrate was neutralized with an aqueous solution of $NaHCO_3$. The product was extracted with CH_2Cl_2, and then washed with brine and dried over anhydrous Na_2SO_4. After filtration and evaporation, the crude product was purified by column chromatography on silica gel (eluent: hexane/CH_2Cl_2 = 2:1, v/v). After removal of the solvents by evaporation, the resulting solid was washed with MeOH to afford **MPASi** as a white solid (yield = 581 mg, 39%). ^{1}H NMR (400 MHz, DMSO-d_6): *δ* 9.23 (s, 1H), 7.48–7.43 (m, 4H), 7.41–7.36 (m, 6H), 7.19 (s, 2H), 7.15 (dd, *J* = 8.4, 1.6 Hz, 2H), 6.97 (d, *J* = 8.4 Hz, 2H), 2.19 (s, 6H). MS (MALDI-TOF): *m*/*z* calcd 377.16 $[M]^+$; found 377.04.

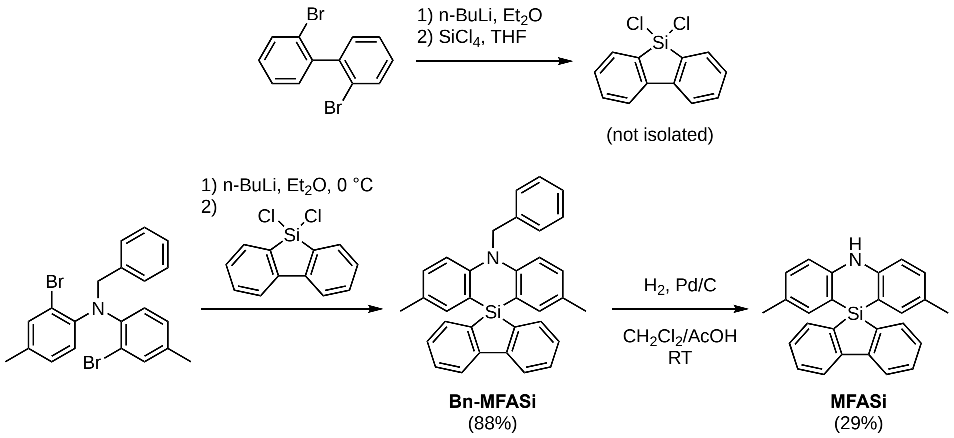

5'-Benzyl-2',8'-dimethyl-5'*H*-spiro[dibenzo[*b,d*]silole-5,10'-dibenzo[*b,e*][1,4]azasiline] (Bn-MFASi): 5,5-Dichloro-5*H*-dibenzo[*b,d*]silole was synthesized according to the literature.[3] To a stirred solution of 2,2'-dibromobiphenyl (2.50 g, 8.00 mmol) in anhydrous diethyl ether (20 mL) was added dropwise *n*-buthyllithium in hexane (1.6 M, 10.3 mL, 16.5 mmol) at −78 °C under N_2. The mixture was stirred at the same temperature for 1 h, and then, stirred at 0 °C for 1 h. The dilithiated intermediate was added dropwise to a stirred solution of tetrachlorosilane (4.5 mL, 40 mmol) precooled to −78 °C in anhydrous THF (40 mL). The reaction mixture was

stirred at the same temperature for 1 h, and allowed to gradually warm to room temperature. After the solvent was removed in vacuo, anhydrous toluene (20 mL) was added and the suspension was vigorously stirred. The inorganic salts were removed by filtration and solvent was removed in vacuo. The crude product was used for the next reaction without purification. To a stirred solution of *N,N*-bis(2-bromo-4-methylphenyl)benzylamine (3.21 g, 7.20 mmol) in anhydrous diethyl ether (40 mL) was added dropwise *n*-buthyllithium in hexane (1.6 M, 9.5 mL, 15.2 mmol) at 0 °C under N_2. The mixture was stirred at 0 °C for 1 h, and then, 5,5-dichloro-5*H*-dibenzo[*b,d*]silole (8.00 mmol, if pure) in anhydrous toluene (12 mL) was added dropwise. The reaction mixture was stirred at 35 °C for 2 h and then cooled to room temperature. After the addition of water, the product was extracted with AcOEt. The combined organic layers were washed with brine and dried over anhydrous Na_2SO_4. After filtration and evaporation, the crude product was purified by column chromatography on silica gel (eluent: hexane/toluene = 4:1, v/v) to afford **Bn-MFASi** as a white solid (yield = 2.94 g, 88%). ^{1}H NMR (400 MHz, $CDCl_3$): δ 7.94 (d, J = 8.0 Hz, 4H), 7.54–7.48 (m, 4H), 7.43–7.32 (m, 5H), 7.24 (d, J = 7.2 Hz, 2H), 7.08 (dd, J = 8.4, 2.0 Hz, 2H), 6.98 (d, J = 2.0 Hz, 2H), 6.92 (d, J = 8.4 Hz, 2H), 5.35 (s, 2H), 2.11 (s, 6H). MS (MALDI-TOF): *m*/*z* calcd 465.19 $[M]^+$; found 465.36.

2',8'-Dimethyl-5'*H*-spiro[dibenzo[*b,d*]silole-5,10'-dibenzo[*b,e*][1,4]azasiline] (MFASi): A suspension of **Bn-MFASi** (1.40 g, 3.00 mmol) and 5% Pd/C (321 mg) in a mixed solvent of CH_2Cl_2 (7.5 mL) and AcOH (7.5 mL) was vigorously stirred under H_2 atmosphere at room temperature for 15 h. After the reaction mixture was filtered through a Celite® pad, the filtrate was neutralized with an aqueous solution of $NaHCO_3$. The product was extracted with CH_2Cl_2, and then washed with brine and dried over anhydrous Na_2SO_4. After filtration and evaporation, the crude product was purified by column chromatography on silica gel (eluent: hexane/CH_2Cl_2 = 2:1, v/v) to afford **MFASi** as a white solid (yield = 322 mg, 29%). ^{1}H NMR (400 MHz, DMSO-d_6): δ 9.50 (s, 1H), 8.05 (d, J = 8.0 Hz, 2H),7.51 (dd, J = 7.6, 7.6 Hz, 2H), 7.34 (d, J = 6.4 Hz, 2H), 7.23 (dd, J = 7.2, 7.2 Hz, 2H), 7.17 (dd, J = 8.8, 2.0 Hz, 2H), 7.06 (d, J = 8.4 Hz, 2H), 6.66 (d, J = 1.6 Hz, 2H), 2.05 (s, 6H). MS (MALDI-TOF): *m*/*z* calcd 375.14 $[M]^+$; found 375.11.

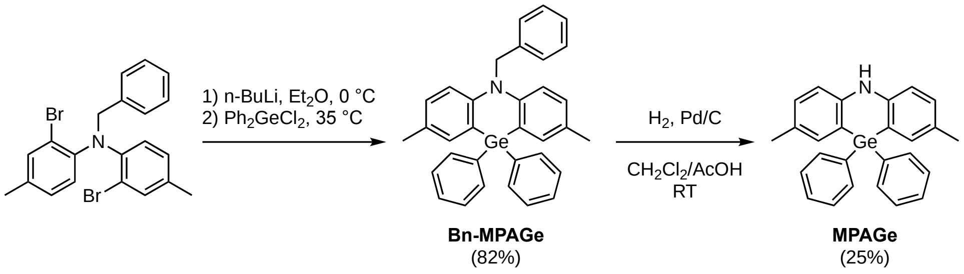

***N*-Benzyl-2,8-dimethyl-10,10-diphenyl-5,10-dihydrodibenzo[*b,e*][1,4]azagermine (Bn-MPAGe):** To a stirred solution of *N*,*N*-bis(2-bromo-4-methylphenyl)benzylamine (2.23 g, 5.00 mmol) in anhydrous diethyl ether (25 mL) was added dropwise *n*-buthyllithium in hexane (1.6 M, 6.6 mL, 10.6 mmol) at 0 °C under N_2. The mixture was stirred at 0 °C for 1 h, and then, dichrorodiphenylgermane (1.65 g, 5.55 mmol) was added dropwise. The reaction mixture was stirred at 35 °C for 2 h and then cooled to room temperature. After the addition of water, the product was extracted with AcOEt. The combined organic layers were washed with brine and dried over anhydrous Na_2SO_4. After filtration and evaporation, the crude product was purified by column chromatography on silica gel (eluent: hexane/CH_2Cl_2 = 4:1, v/v). After removal of the solvents by evaporation, the resulting solid was washed with MeOH to afford **Bn-MPAGe** as a white solid (yield = 2.11 g, 82%). ^{1}H NMR (400 MHz, $CDCl_3$): δ 7.56–7.53 (m, 4H), 7.42–7.36 (m, 6H), 7.32–7.27 (m, 5H), 7.24–7.19 (m, 5H), 7.14 (d, *J* = 7.6 Hz, 2H), 7.03 (dd, *J* = 8.4, 2.0 Hz, 2H), 6.90 (d, *J* = 8.8 Hz, 2H), 5.12 (s, 2H), 2.23 (s, 6H). MS (MALDI-TOF): *m*/*z* calcd 512.15 $[M]^+$; found 512.37.

2,8-Dimethyl-10,10-diphenyl-5,10-dihydrodibenzo[*b,e*][1,4]azagermine (MPAGe): A suspension of **Bn-MPAGe** (1.54 g, 3.00 mmol) and 5% Pd/C (320 mg) in a mixed solvent of CH_2Cl_2 (7.5 mL) and AcOH (7.5 mL) was vigorously stirred under H_2 atmosphere at room temperature for 24 h. After the reaction mixture was filtered through a Celite® pad, the filtrate was neutralized with an aqueous solution of $NaHCO_3$. The product was extracted with CH_2Cl_2, and then washed with brine and dried over anhydrous Na_2SO_4. After filtration and evaporation, the crude product was purified by column chromatography on silica gel (eluent: hexane/CH_2Cl_2 = 2:1, v/v). After removal of the solvents by evaporation, the resulting solid was washed with MeOH to afford **MPAGe** as a white solid (yield = 319 mg, 25%). ^{1}H NMR (400 MHz, DMSO-d_6): δ 8.99 (s, 1H), 7.47–7.43 (m, 4H), 7.42–7.38 (m, 6H), 7.17 (d, *J* = 1.2 Hz, 2H), 7.10 (dd, *J* = 8.4, 2.4 Hz, 2H), 6.96 (d, *J* = 8.4 Hz, 2H), 2.19 (s, 6H). MS (MALDI-TOF): *m*/*z* calcd 4232.10 $[M]^+$; found 423.10.

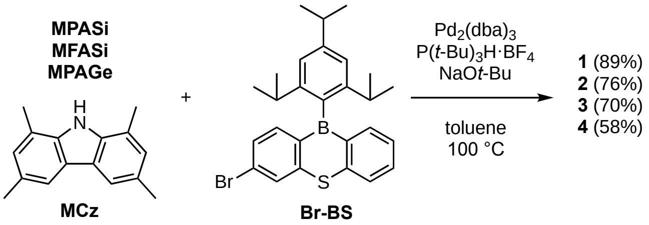

Compound 1 (MPASi-BS): A mixture of **MPASi** (453 mg, 1.20 mmol), **Br-BS** (573 mg, 1.20 mmol), $Pd_2(dba)_3$ (22 mg, 0.024 mmol), P(*t*-Bu)$_3$H·BF_4 (28 mg, 0.097 mmol), and sodium *tert*-butoxide (233 mg, 2.43 mmol) in anhydrous toluene (12 mL) was stirred at 100 °C for 14 h under N_2. After addition of aqueous solution of NH_4Cl, the product was extracted with toluene. The combined organic layer was washed with brine, dried over anhydrous Na_2SO_4, and concentrated under reduced pressure. The crude product was purified by column chromatography on silica gel (eluent: hexane/toluene = 5:1, v/v) to afford **1** as a yellow solid (yield = 828 mg, 89%). ^{1}H NMR (400 MHz, $CDCl_3$): δ 7.93–7.90 (m, 2H), 7.70 (d, J = 7.6 Hz, 1H), 7.60–7.56 (m, 5 H), 7.45 (d, J = 1.6 Hz, 1H), 7.39–7.28 (m, 9H), 7.07 (s, 2H), 7.06–7.01 (m, 3H), 6.67 (d, J = 8.4 Hz, 1H), 2.99 (sept, J = 6.8 Hz, 1H), 2.29 (sept, J = 6.8 Hz, 2H), 2.25 (s, 6H), 1.35 (d, J = 6.8 Hz, 6H), 1.01 (d, J = 6.8 Hz, 12H). ^{13}C NMR (100 MHz, $CDCl_3$): δ 149.99, 148.32, 148.03, 147.46, 145.60, 143.26, 142.36, 140.20, 136.15, 135.90, 134.21, 131.82, 131.17, 130.86, 129.61, 127.84, 124.91, 124.40, 122.59, 121.53, 121.30, 120.21, 120.03, 35.37, 34.27, 24.31, 24.27, 24.20, 20.54. MS (MALDI-TOF): calcd 773.37 $[M]^+$; found 773.00. Anal. calcd (%) for $C_{53}H_{52}BNSSi$: C 82.25, H 6.77, N 1.81; found: C 81.78, H 6.73, N 1.83. T_d = 368 °C.

Compound 2 (MFASi-BS): A mixture of **MFASi** (376 mg, 1.00 mmol), **Br-BS** (478 mg, 1.00 mmol), $Pd_2(dba)_3$ (18 mg, 0.020 mmol), P(*t*-Bu)$_3$H·BF_4 (24 mg, 0.081 mmol), and sodium *tert*-butoxide (192 mg, 2.00 mmol) in anhydrous toluene (10 mL) was stirred at 100 °C for 17 h under N_2. After addition of aqueous solution of NH_4Cl, the product was extracted with toluene. The combined organic layer was washed with brine, dried over anhydrous Na_2SO_4, and concentrated under reduced pressure. The crude product was purified by column chromatography on silica gel (eluent: hexane/toluene = 4:1, v/v) to afford **2** as a yellow solid (yield = 589 mg, 76%). ^{1}H NMR (400 MHz, $CDCl_3$): δ 8.08 (d, J = 8.0 Hz, 1H), 7.97 (dd, J = 7.6, 1.2 Hz, 1H), 7.93 (d, J = 8.0 Hz, 2H), 7.74 (d, J = 8.0 Hz, 2H), 7.65 (d, J = 2.0 Hz, 1H), 7.61 (ddd, J = 8.0, 8.0, 1.2 Hz, 1H), 7.49 (ddd, J = 8.0, 8.0, 1.6 Hz, 2H), 7.42 (d, J = 7.2 Hz, 2H), 7.34 (ddd, J = 8.0, 8.0, 1.2 Hz, 1H), 7.22–7.18 (m, 3H), 7.10 (s, 2H), 7.07–7.04 (m, 4H), 6.70 (m, 2H), 3.00 (sept, J = 6.8 Hz, 1H), 2.35 (sept, J = 6.8 Hz, 2H), 2.13 (s, 6H), 1.37 (d, J = 6.8 Hz, 6H), 1.05 (d, J = 6.8 Hz, 6H), 1.05 (d, J = 6.8 Hz, 6H). ^{13}C NMR (100 MHz, $CDCl_3$):

δ 150.03, 148.93, 148.44, 148.30, 146.05, 143.25, 142.80, 136.45, 135.56, 134.51, 131.98, 131.84, 131.01, 130.75, 127.92, 124.98, 124.54, 122.89, 121.90, 120.85, 120.29, 120.11, 118.46, 35.50, 34.29, 24.32, 24.27, 24.20, 20.28. MS (MALDI-TOF): calcd 771.35 $[M]^+$; found 771.20. Anal. calcd (%) for $C_{53}H_{50}BNSSi$: C 82.46, H 6.53, N 1.81; found: C 82.41, H 6.43, N 1.88. T_d = 380 °C.

Compound 3 (MPAGe-BS): A mixture of **MPAGe** (422 mg, 1.00 mmol), **Br-BS** (477 mg, 1.20 mmol), $Pd_2(dba)_3$ (19 mg, 0.021 mmol), P(*t*-Bu)$_3$H·BF$_4$ (24 mg, 0.082 mmol), and sodium *tert*-butoxide (194 mg, 2.01 mmol) in anhydrous toluene (10 mL) was stirred at 100 °C for 16 h under N_2. After addition of aqueous solution of NH_4Cl, the product was extracted with toluene. The combined organic layer was washed with brine, dried over anhydrous Na_2SO_4, and concentrated under reduced pressure. The crude product was purified by column chromatography on silica gel (eluent: hexane/toluene = 4:1, v/v) to afford **3** as a white solid (yield = 573 mg, 70%). ^{1}H NMR (400 MHz, $CDCl_3$): δ 7.79 (dd, J = 8.0, 1.6 Hz, 1H), 7.59 (d, J = 7.6 Hz, 1H), 7.48 (ddd, J = 8.0, 8.0, 1.6 Hz, 1H), 7.44 (d, J = 8.4 Hz, 1H), 7.41–7.38 (m, 6H), 7.31 (d, J = 2.0 Hz, 2H), 7.24–7.17 (m, 9H), 7.00 (s, 2H), 6.93 (d, J = 2.4 Hz, 1H), 6.64 (dd, J = 8.8, 2.4 Hz, 1H), 2.94 (sept, J = 6.8 Hz, 1H), 2.33 (s, 6H), 2.24 (sept, J = 6.8 Hz, 2H), 1.32 (d, J = 7.2 Hz, 6H), 0.96 (d, J = 6.8 Hz, 6H), 0.95 (d, J = 6.8 Hz, 6H). ^{13}C NMR (100 MHz, $CDCl_3$): δ 150.36, 149.95, 147.80, 145.60, 145.08, 143.25, 141.18, 139.73, 135.66, 135.63, 135.20, 133.53, 131.00, 130.53, 129.25, 128.09, 127.04, 124.64, 123.91, 119.72, 112.88, 109.19, 34.97, 34.26, 24.42, 24.36, 24.20, 20.95. MS (MALDI-TOF): calcd 819.31 $[M]^+$; found 819.10. Anal. calcd (%) for $C_{53}H_{52}BGeNS$: C 77.77, H 6.40, N 1.71; found: C 77.56, H 6.43, N 1.74. T_d = 371 °C.

Compound 4 (MCz-BS): A mixture of **MCz** (493 mg, 2.21 mmol), **Br-BS** (955 mg, 2.00 mmol), $Pd_2(dba)_3$ (37 mg, 0.040 mmol), P(*t*-Bu)$_3$H·BF$_4$ (46 mg, 0.16 mmol), and sodium *tert*-butoxide (302 mg, 3.96 mmol) in anhydrous toluene (20 mL) was stirred at 100 °C for 6 h under N_2. After addition of aqueous solution of NH_4Cl, the product was extracted with toluene. The combined organic layer was washed with brine, dried over anhydrous Na_2SO_4, and concentrated under reduced pressure. The crude product was purified by column chromatography on silica gel (eluent: hexane/toluene = 5:1, v/v) to afford **4** as a white solid (yield = 712 mg, 58%). ^{1}H NMR (400 MHz, $CDCl_3$): δ 8.02 (dd, J = 8.0, 1.2 Hz, 1H), 7.97 (d, J = 8.0 Hz, 1H), 7.83 (d, J = 2.0 Hz, 1H), 7.77–7.75 (m, 3H), 7.63 (ddd, J = 8.0, 8.0, 1.6 Hz, 1H), 7.42 (dd, J = 8.0, 2.0 Hz, 1H), 7.36 (ddd, J = 8.0, 8.0, 0.8 Hz, 1H), 7.10 (s, 2H), 6.91 (s, 2H), 3.00 (sept, J = 6.8 Hz, 1H), 2.47 (s, 6H), 2.28 (sept, J = 6.8 Hz, 2H), 1.86 (s, 6H), 1.36 (d, J = 6.8 Hz, 6H), 1.04 (d, J

= 6.8 Hz, 6H), 0.99 (d, J = 6.8 Hz, 6H). ^{13}C NMR (100 MHz, $CDCl_3$): δ 149.95, 148.64, 145.62, 143.48, 143.23, 140.40, 140.28, 139.40, 137.57, 134.70, 132.30, 130.29, 129.25, 127.39, 127.22, 125.07, 124.76, 124.34, 121.20, 120.18, 117.85, 35.61, 34.28, 24.19, 24.12, 24.08, 21.10, 19.30. MS (MALDI-TOF): calcd 619.34 $[M]^+$; found 619.24. Anal. calcd (%) for $C_{43}H_{46}BNS$: C 83.34, H 7.48, N 2.26; found: C 83.39, H 7.41, N 2.28. T_d = 343 °C.

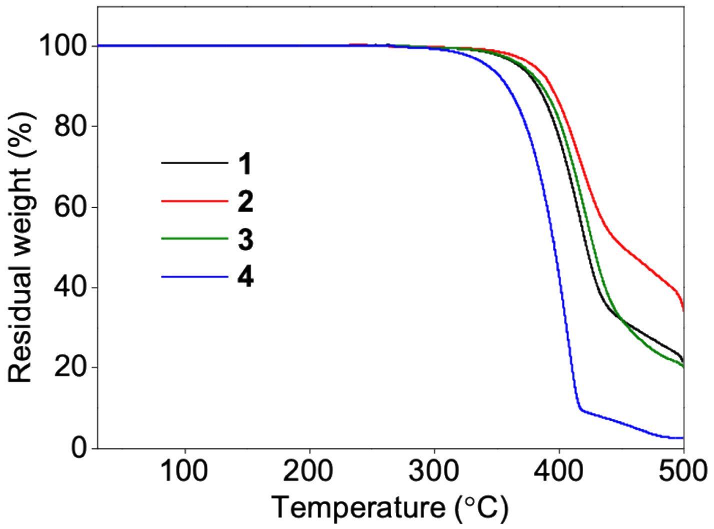

Fig. S1 TGA thermograms of **1–4** at a heating rate of 10 °C min^{-1} under N_2.

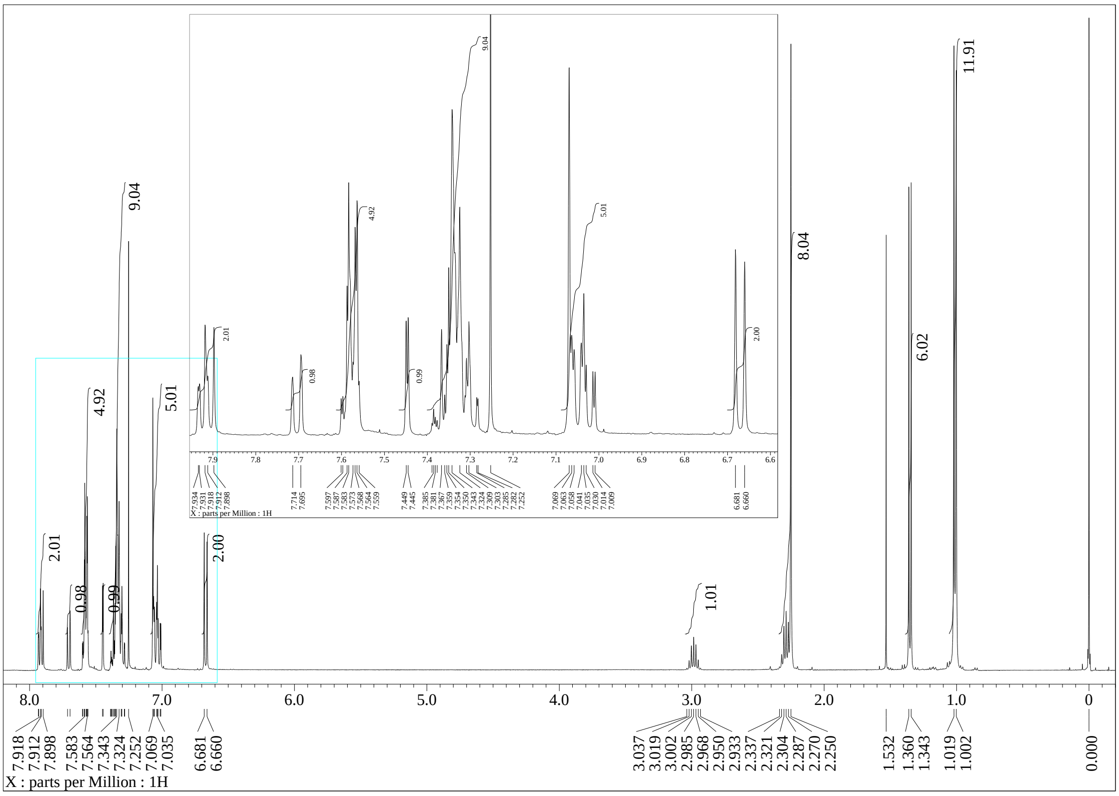

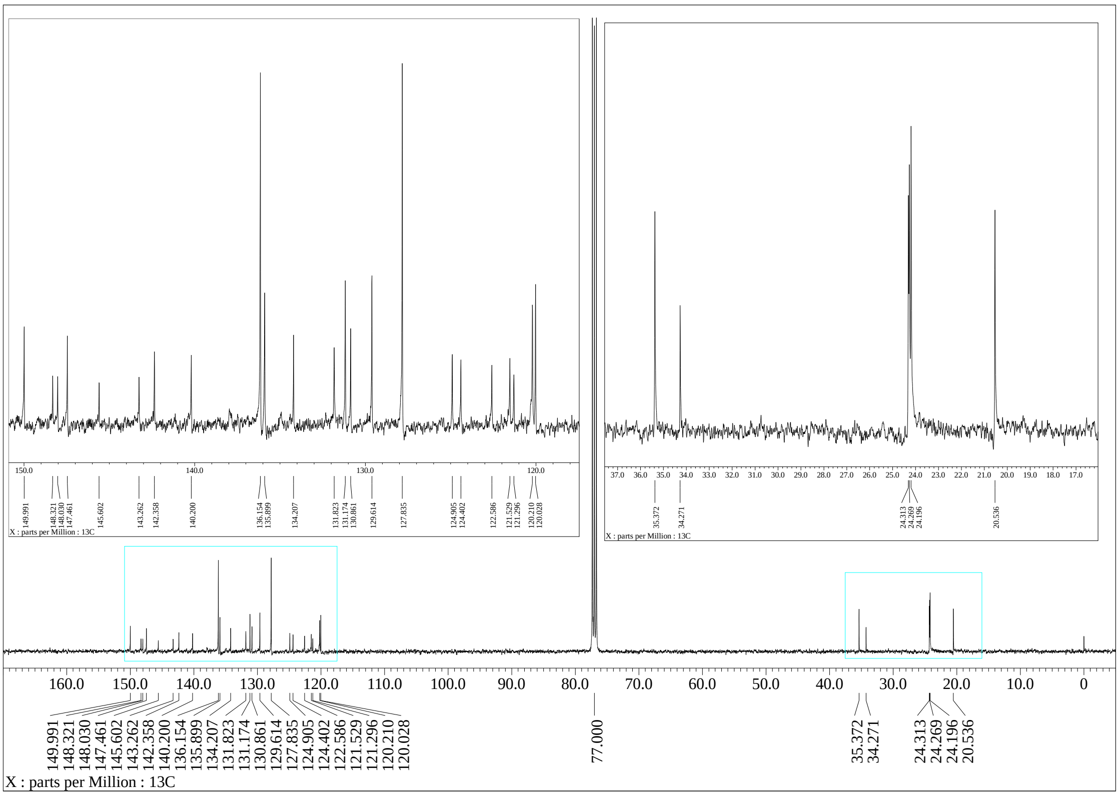

Fig. S2 ^{1}H and ^{13}C NMR spectra of **1** in $CDCl_3$.

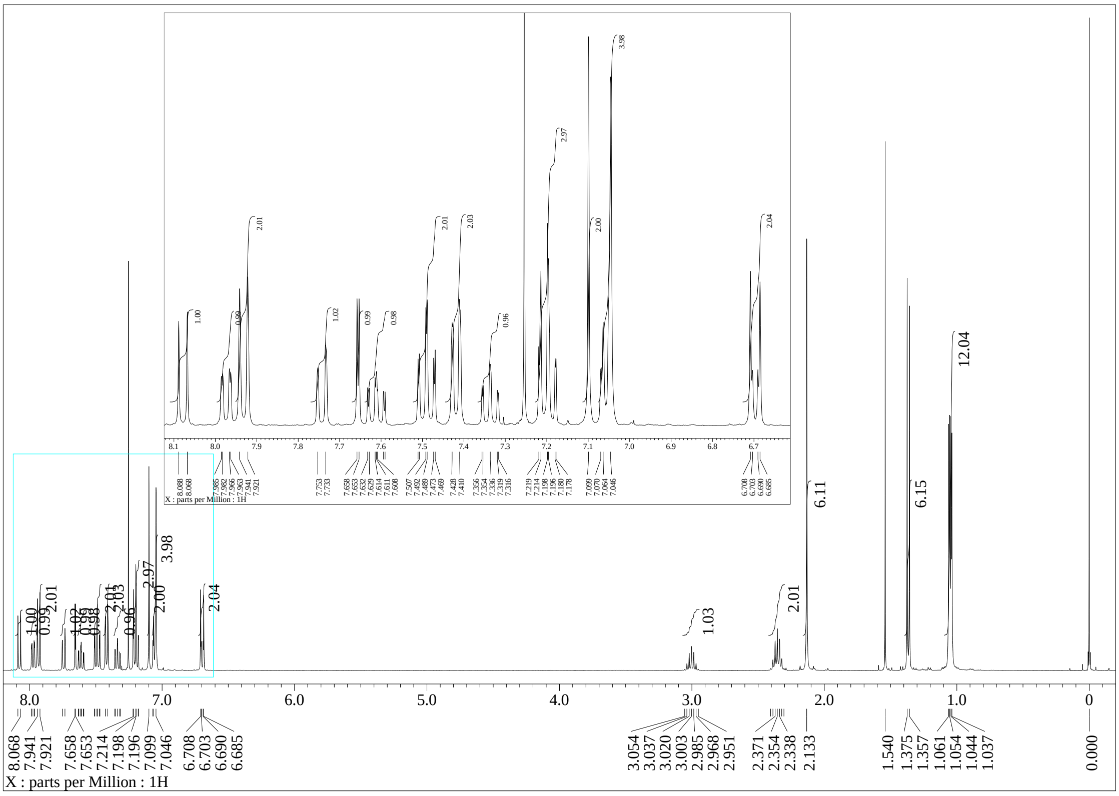

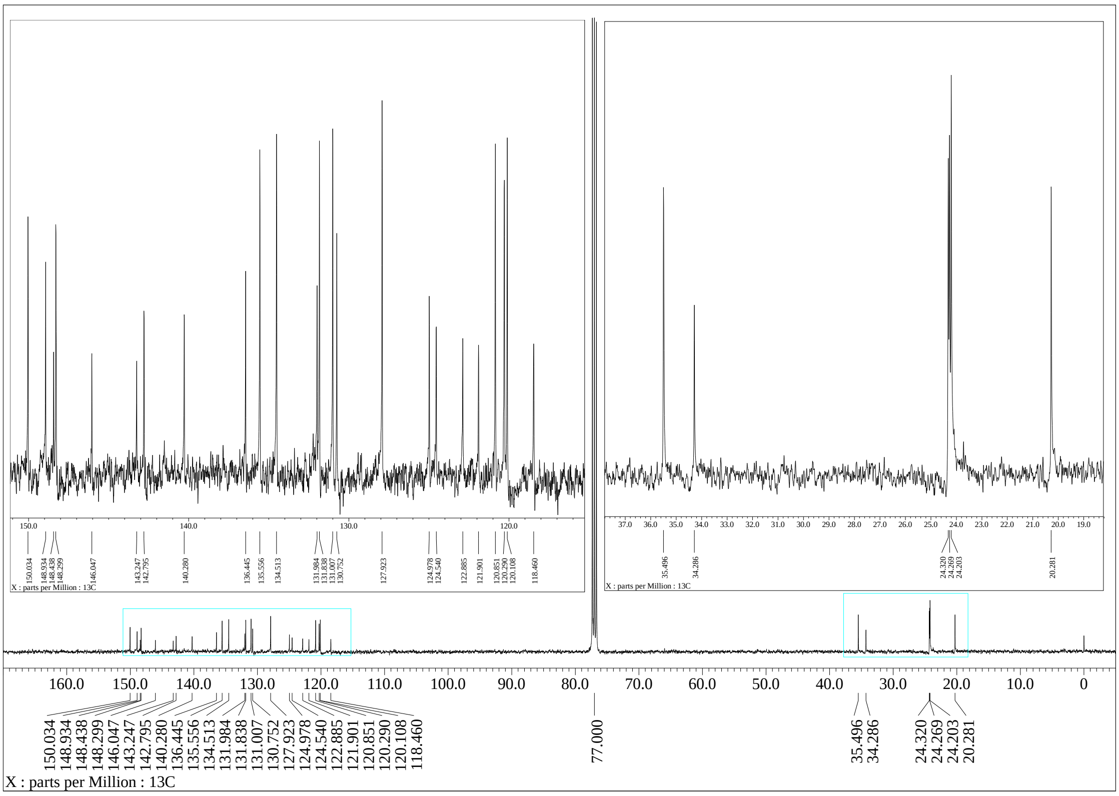

Fig. S3 ^{1}H and ^{13}C NMR spectra of **2** in $CDCl_3$.

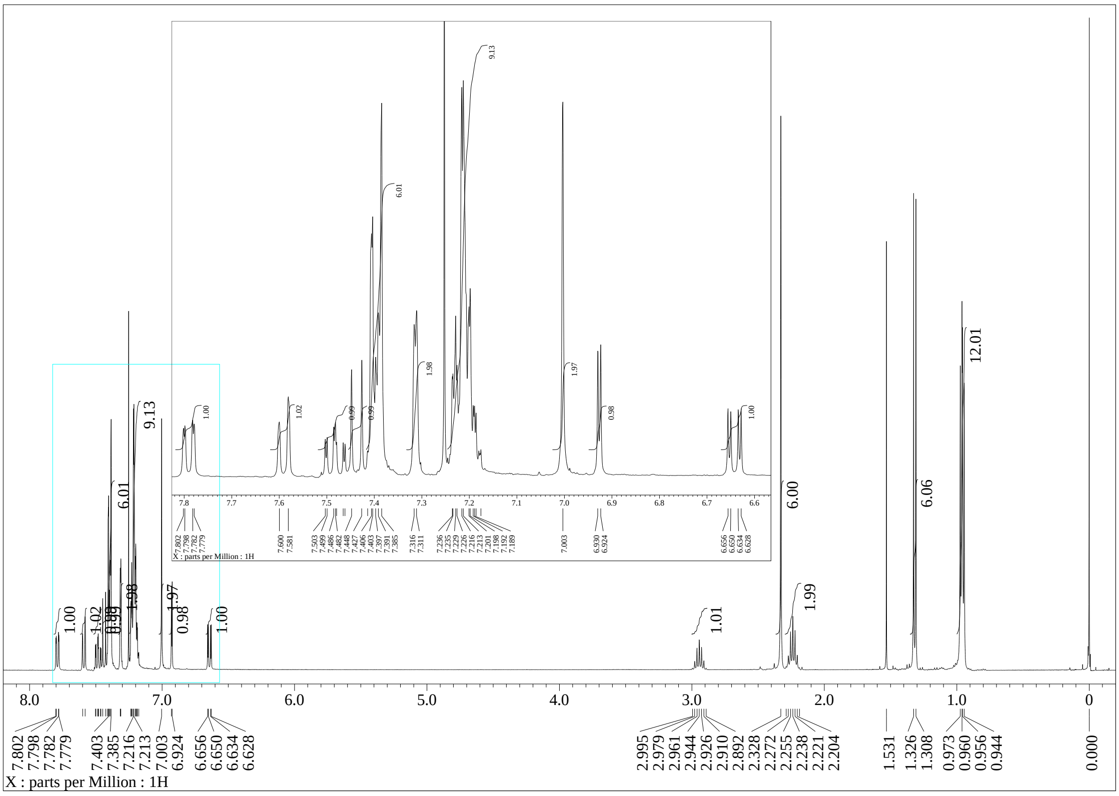

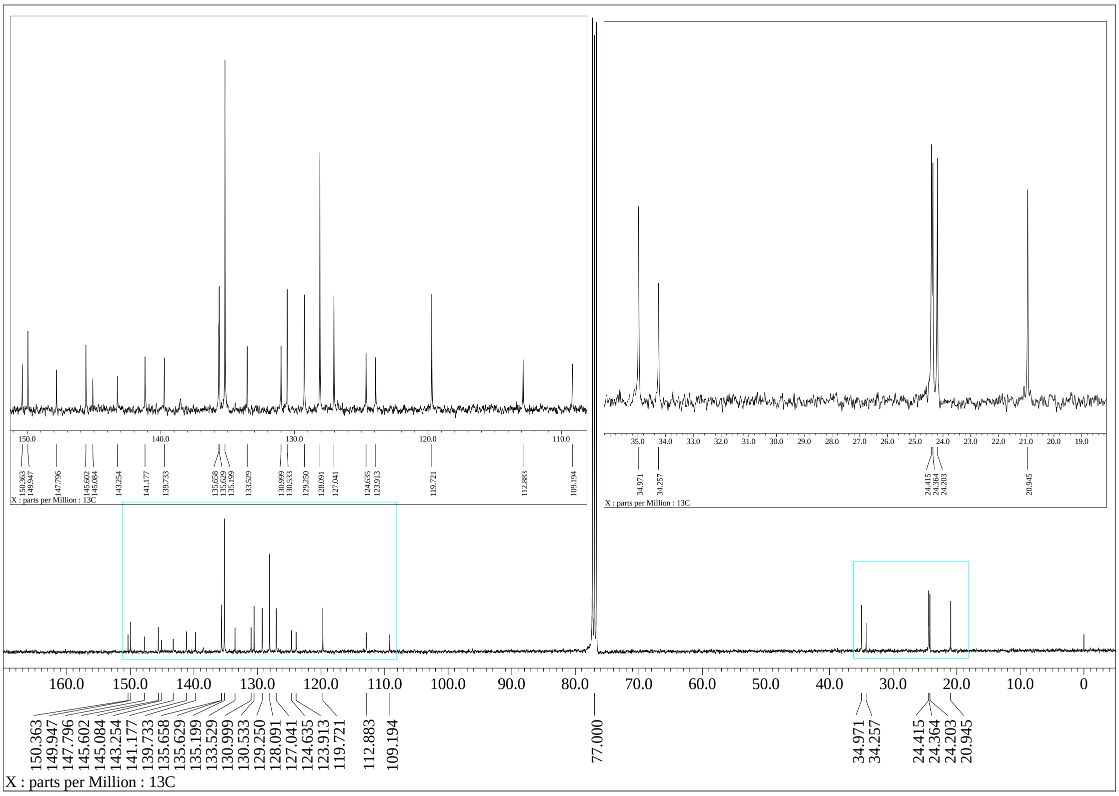

Fig. S4 ^{1}H and ^{13}C NMR spectra of **3** in $CDCl_3$.

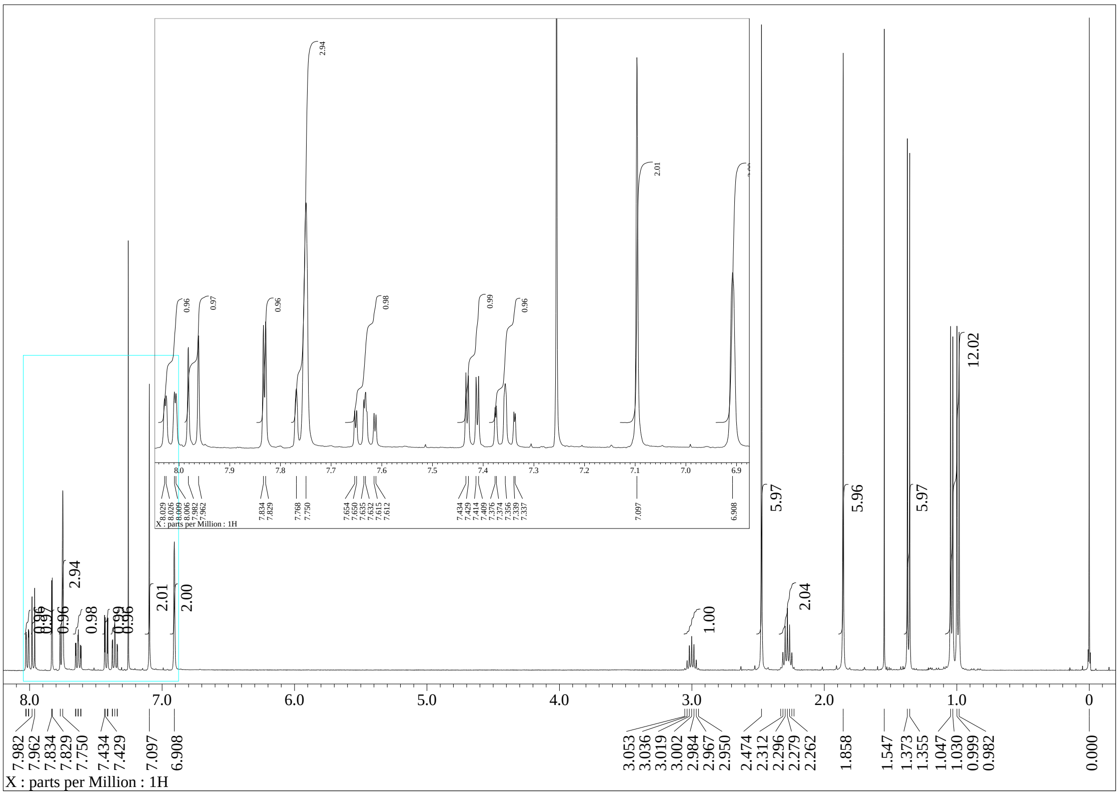

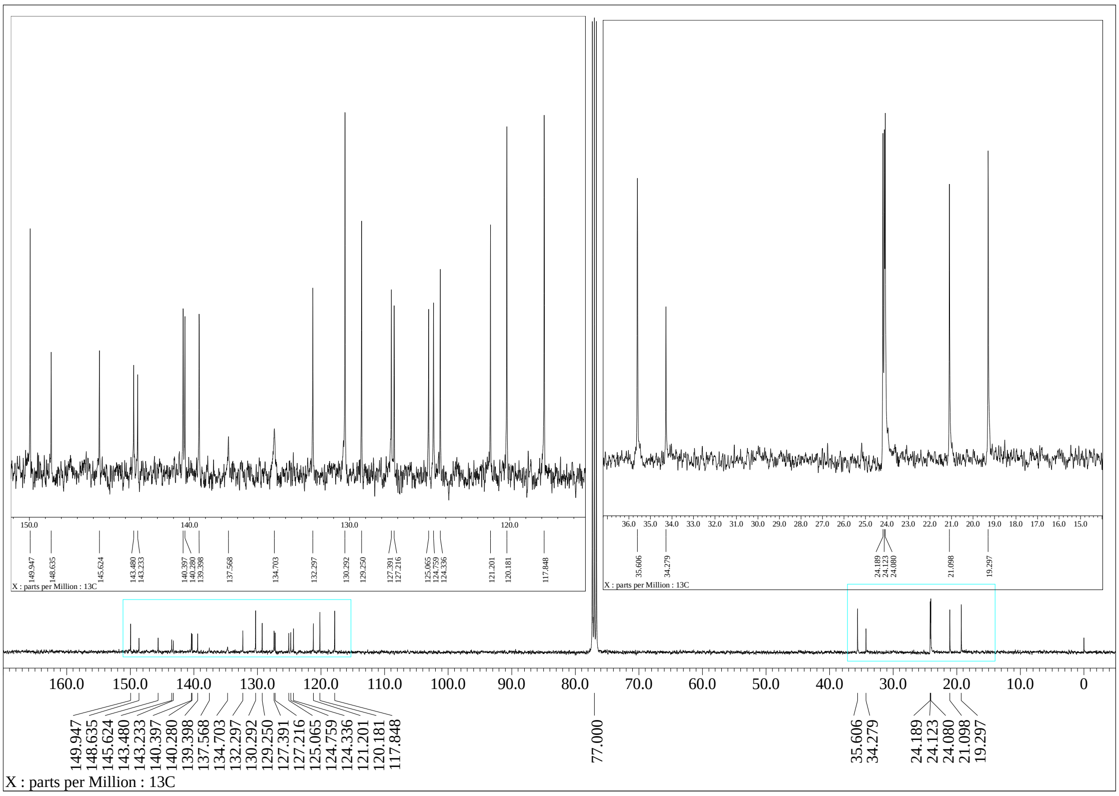

Fig. S5 ^{1}H and ^{13}C NMR spectra of **4** in $CDCl_3$.

2. X-ray Crystallographic Analysis

Single crystal X-ray diffraction measurements were performed on a Rigaku AFC-10 diffractometer with a Saturn724+ CCD detector for **1** and **4**, and a Rigaku XtaLAB diffractometer with a HyPix-6000 detector for **2** and **3**. The diffraction data were collected using Mo-Kα radiation (λ = 0.71073 Å) monochromated with multi-layer mirror. The structures were solved by direct methods (SHELXT-2016[4]) and refined by the full-matrix least-squares on F^2 (SHELXL-2016[4]). All non-hydrogen atoms were refined anisotropically. All hydrogen atoms were placed using AFIX instructions.

Crystallographic data for 1: The measurement was performed at 163 K. Total 46622 reflections were collected, among which 10032 reflections were independent (R_{int} = 0.0892). The crystal data: Formula $C_{53}H_{52}BNSSi$; FW = 773.91, crystal size 0.13 × 0.08 × 0.05 mm, Monoclinic, $P2_1/n$ (#14), a = 16.28(3) Å, b = 8.916(15) Å, c = 30.47(5) Å, $\alpha = \gamma$ = 90°, β = 97.92(3)°, V = 4381(13) Å^3, Z = 4, D_{calcd} = 1.173 g cm^{-3}, R_1 = 0.0681 ($I > 2\sigma(I)$), wR_2 = 0.1773 (all data), GOF = 1.068. Two isopropyl groups were disordered. The crystal data is deposited in The Cambridge Crystallographic Data Centre (CCDC number: 1948037).

Crystallographic data for 2: The measurement was performed at 173 K. Total 50514 reflections were collected, among which 10707 reflections were independent (R_{int} = 0.0343). The crystal data: Formula $C_{53}H_{50}BNSSi·C_4H_8O_2$; FW = 860.00, crystal size 0.16 × 0.15 × 0.06 mm, Monoclinic, $P2_1/c$ (#14), a = 18.0660(3) Å, b = 13.8091(3) Å, c = 19.3607(3) Å, $\alpha = \gamma$ = 90°, β = 99.953(2)°, V = 4757.32(15) Å^3, Z = 4, D_{calcd} = 1.201 g cm^{-3}, R_1 = 0.0610 ($I > 2\sigma(I)$), wR_2 = 0.1803 (all data), GOF = 1.039. One AcOEt molecule used as solvent were contained in an asymmetric unit of the crystal lattice. One isopropyl group was disordered. The crystal data is deposited in The Cambridge Crystallographic Data Centre (CCDC number: 1948038).

Crystallographic data for 3: The measurement was performed at 173 K. Total 90630 reflections were collected, among which 10418 reflections were independent (R_{int} = 0.0431). The crystal data: Formula $C_{53}H_{52}BNSGe·C_2H_3N$; FW = 859.47, crystal size 0.25 × 0.13 × 0.08 mm, Monoclinic, $P2_1/n$ (#14), a = 14.0919(2) Å, b = 9.10970(10) Å, c = 36.2632(6) Å, $\alpha = \gamma$ = 90°, β = 100.447(2)°, V = 4578.04(12) Å^3, Z = 4, D_{calcd} = 1.247 g cm^{-3}, R_1 = 0.0339 ($I > 2\sigma(I)$), wR_2 = 0.0993 (all data), GOF = 1.086. One acetonitrile molecule used as solvent were contained in an asymmetric unit of the crystal lattice. The crystal data is deposited in The Cambridge Crystallographic Data Centre (CCDC number: 1948039).

Crystallographic data for 4: The measurement was performed at 163 K. Total 17959 reflections were collected, among which 7985 reflections were independent (R_{int} = 0.0657). The crystal data: Formula $C_{43}H_{46}BNS$; FW = 619.68, crystal size 0.15 × 0.05 × 0.03 mm, Triclinic, *P*–1 (#2), a = 10.103(8) Å, b = 12.791(10) Å, c = 13.778(12) Å, α = 84.552(16)°, β = 86.292(18)°, γ = 83.85(2)°, V = 1760(2) $Å^3$, Z = 2, D_{calcd} = 1.170 g cm^{-3}, R_1 = 0.0800 ($I > 2\sigma(I)$), wR_2 = 0.3052 (all data), GOF = 1.065. The crystal data is deposited in The Cambridge Crystallographic Data Centre (CCDC number: 1948040).

3. Electrochemical Analysis

Cyclic voltammetry (CV) and differential pulse voltammetry (DPV) were performed using an ALS CHI 612E electrochemical analyzer and a three-electrode cell equipped with Pt working and counter electrodes and an Ag/$AgNO_3$ reference electrode. The measurements were carried out for CH_2Cl_2 solutions of **1–5** (1 mM) at a scanning rate of 50 mV s^{-1}. Tetrabutylammonium perchlorate (n-Bu_4NClO_4) was used as a supporting electrolyte with a concentration of 0.1 M. The redox potentials were calibrated with ferrocene as an internal standard.

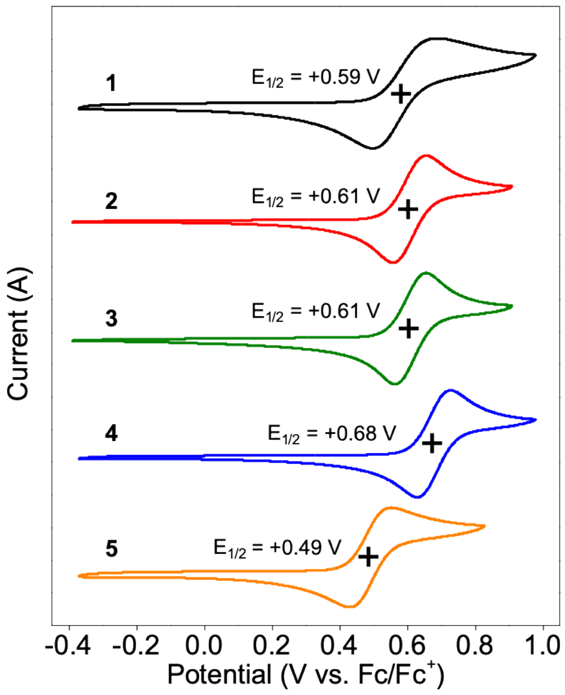

Fig. S6 Cyclic voltammograms of **1–5** in CH_2Cl_2 measured at a scanning rate of 50 mV s^{-1}.

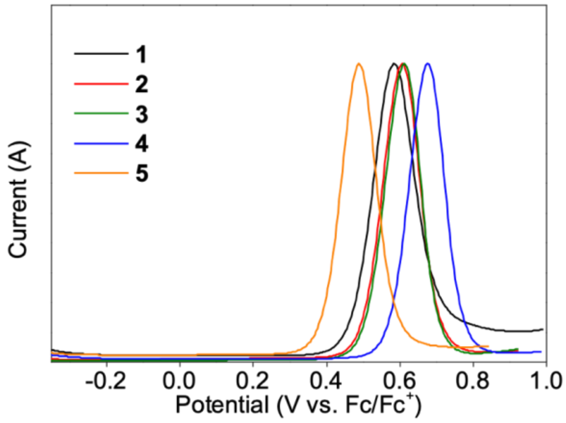

Fig. S7 Differential pulse voltammograms of **1–5** in CH_2Cl_2.

4. Photophysical Properties

UV–vis absorption spectra were measured with a Jasco V-670 spectrometer. Fluorescence and phosphorescence spectra were measured with a Jasco FP-8600 spectrophotometer. Absolute photoluminescence quantum yields (Φ_{PL}) were determined with a Jasco ILF-835 integrating sphere system. The transient PL decay measurements were performed using a Quantaurus-Tau C11367 under N_2. Photophysical properties of **1–4** in toluene solutions were measured by using deoxygenated spectral grade solvents with the concentration of 2~5 × 10^{-5} M. Pure neat films for the optical measurements were deposited under high vacuum (< 1 × 10^{-4} Pa) onto quartz substrates. The optical energy gaps (E_g) were determined from the high-energy onset position of the absorption spectra of pure neat films. The ionization potentials (IP) were determined from the photoelectron yield spectra of pure neat films. The electron affinities (EA) were estimated by subtracting E_g from the measured IP. The 50 wt%-doped films for the optical measurements were co-deposited with PPF as a host material under high vacuum (< 1 × 10^{-4} Pa) onto quartz substrates. The singlet and triplet excitation energies (E_S and E_T) of **1–4** were estimated from the high-energy onset positions of the fluorescence and phosphorescence spectra of the doped films. The phosphorescence spectra of **MPASi**, **MFASi**, **MPAGe**, **MCz**, and **Br-BS** were measured in frozen toluene solutions at 77 K. The phosphorescence spectrum of **Br-BS** in the film was measured using 50 wt%-doped film in PPF prepared by drop-casting. The rate constants for the TADF decay process of **1–4** were calculated according to the literature.[5]

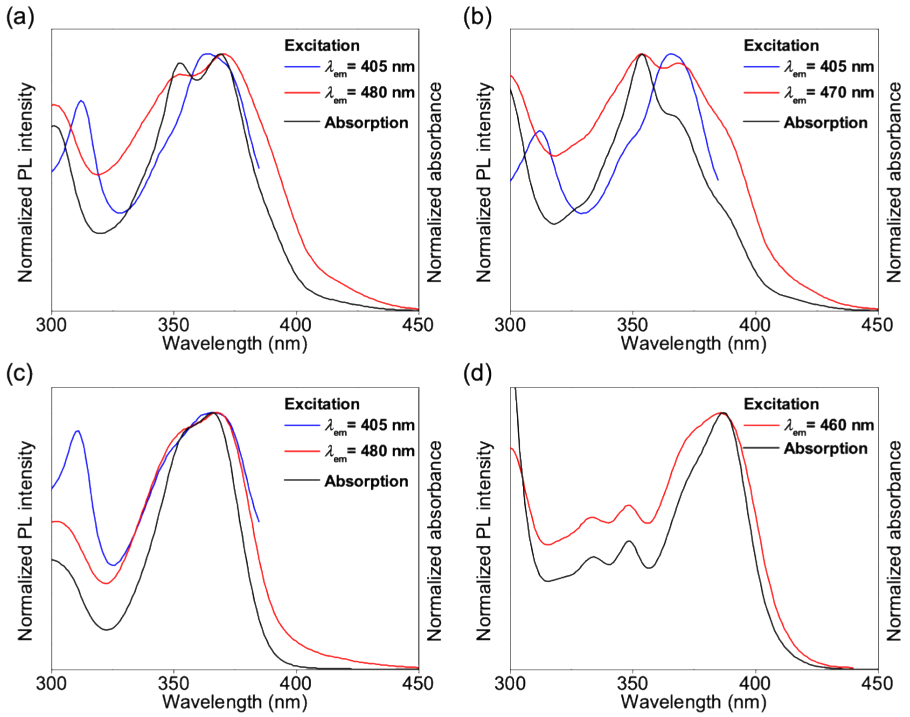

Fig. S8 Absorption and excitation spectra (for QE and QA) of (a) **1**, (b) **2**, (c) **3**, and (d) **4** in toluene solutions.

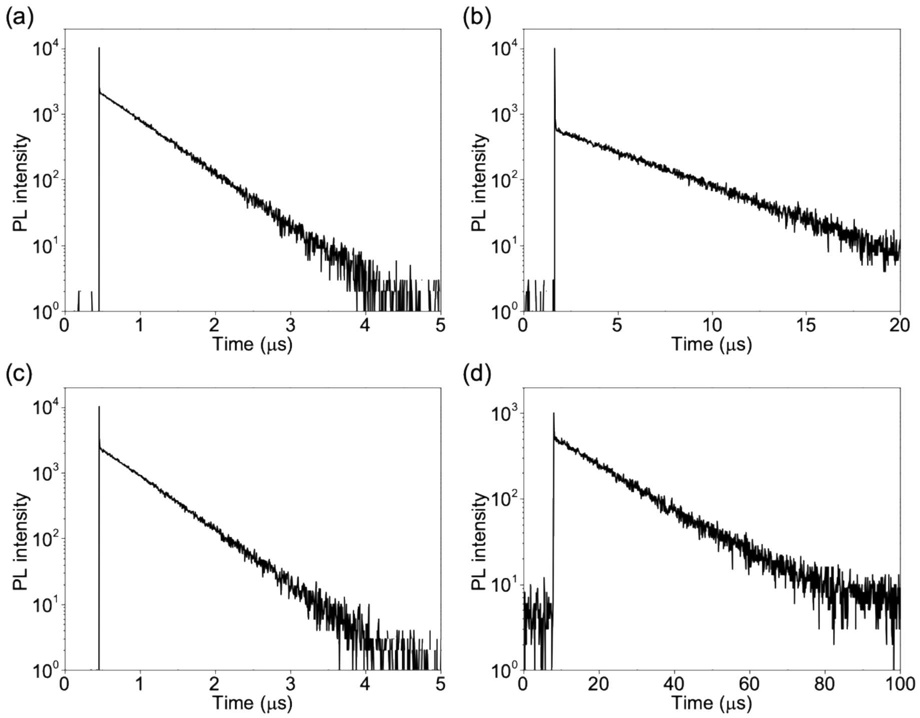

Fig. S9 Transient PL decay curves (for QE) of (a) **1**, (b) **2**, (c) **3**, and (d) **4** in deoxygenated toluene solutions at 300 K.

Table S1 Photophysical data for **1–4** in toluene solutions

	λ_{PL} [nm]	E_{FWHM} [eV]	λ_{FWHM} [nm]	Φ_{PL} [%]	τ_p [ns]	τ_d [µs]
**1**	409, 478	0.36^a	68^a	46^b	1.4^c	0.54^c
**2**	410, 472	0.38^a	69^a	27^b	5.3^c	4.3^c
**3**	403, 482	0.37^a	70^a	42^b	1.9^c	0.53^c
**4**	458	0.38	67	22	0.39	15.7

^{a}Full width at haif-maximum of the PL for QE. ^{b}PL quantum yield evaluated from overall emissions of QA and QE. ^{c}Emission lifetime for QE.

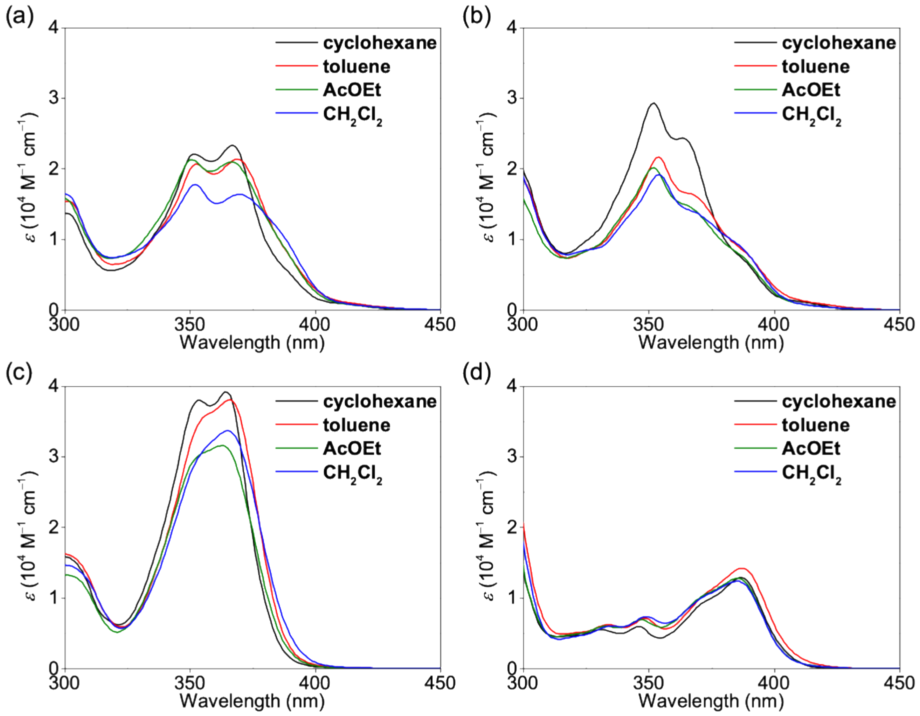

Fig. S10 UV–vis absorption spectra of (a) **1**, (b) **2**, (c) **3**, and (d) **4** in various solvents.

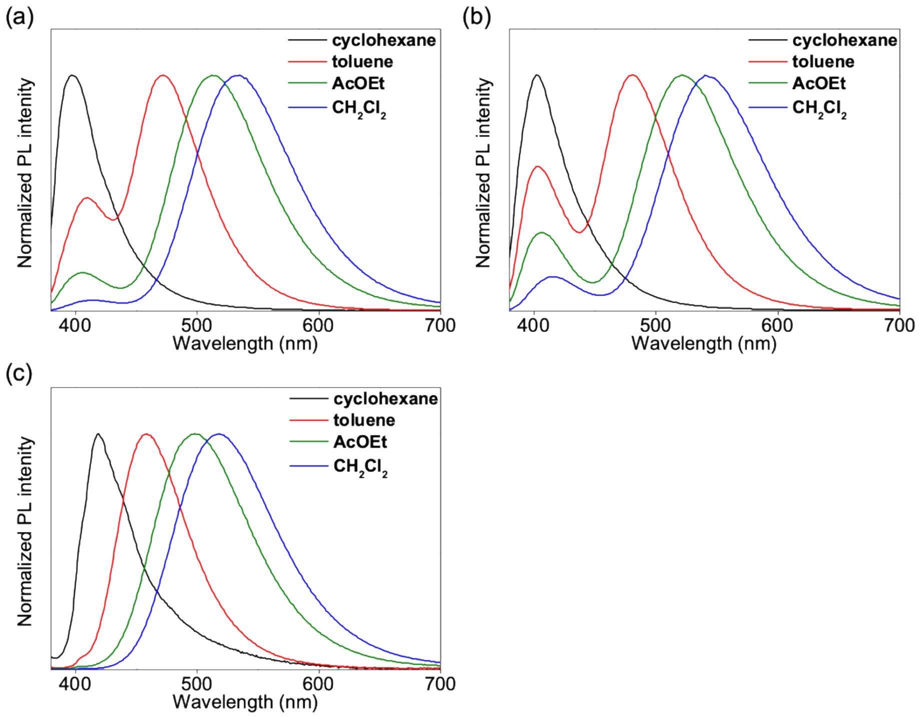

Fig. S11 PL spectra of (a) **2**, (b) **3**, and (c) **4** in various solvents.

Table S2 Summary of the maximum PL wavelength of **1–4** in different solvents

	cyclohexane	toluene	AcOEt	CH_2Cl_2
**1**	403	409, 478	410, 517	419, 540
**2**	397	410, 472	405, 512	413, 535
**3**	403	403,482	407, 521	416, 540
**4**	419	458	498	518

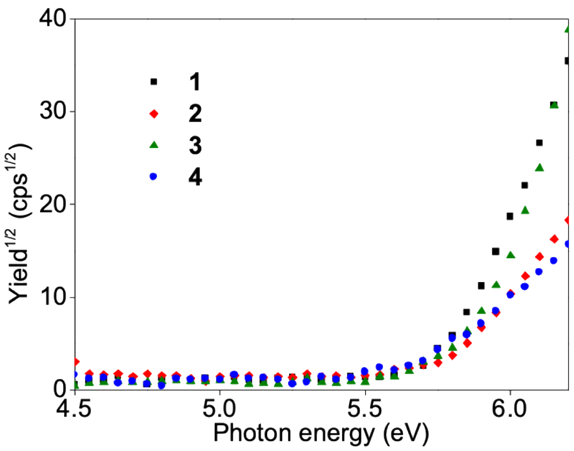

Fig. S12 Photoelectron yield spectra of **1**–**4** in neat films.

Table S3 Photophysical data for **1**–**4** in neat films

	λ_{abs} (onset) [nm]	E_g [eV]	E_{IP} [eV]	E_{EA} [eV]
**1**	440	2.82	5.78	2.96
**2**	438	2.83	5.77	2.94
**3**	391	3.17	5.87	2.70
**4**	429	2.89	5.68	2.79

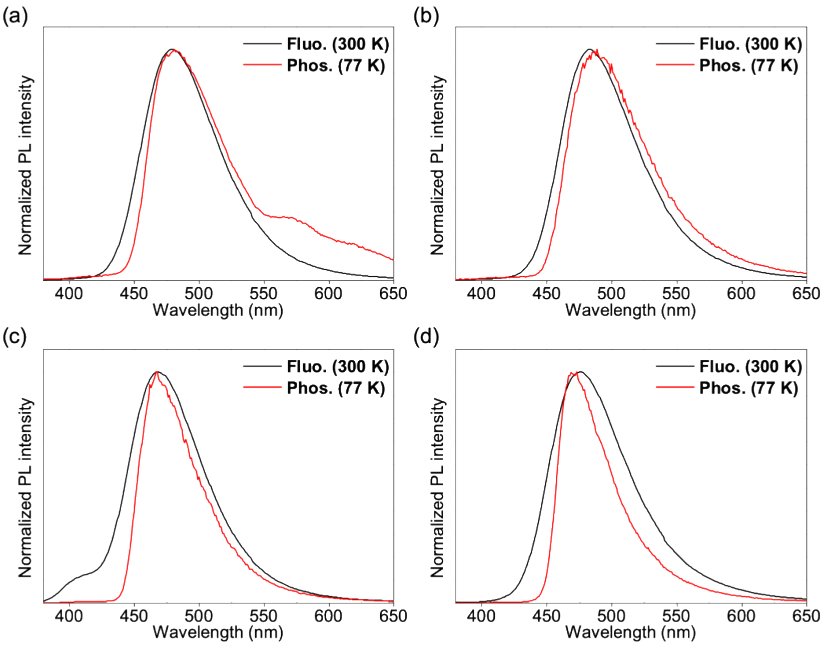

Fig. S13 Fluorescence (300 K) and phosphorescence spectra of (a) **1**, (b) **2**, (c) **3**, and (d) **4** in 50 wt%-doped films.

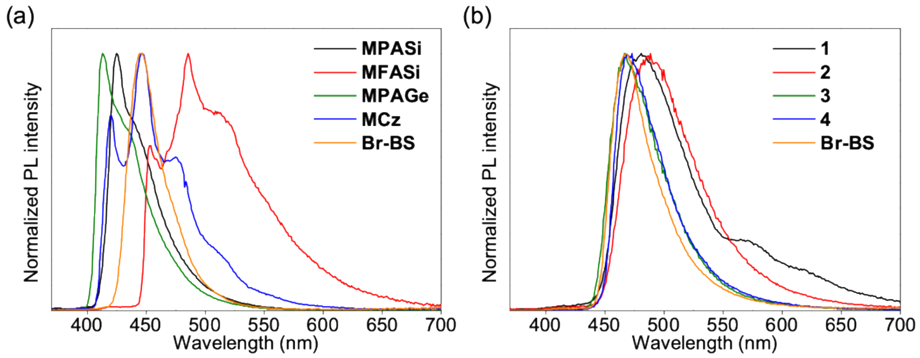

Fig. S14 (a) Phosphorescence spectra of donor and acceptor units in frozen toluene solutions at 77 K. (b) Phosphorescence spectra of **1–4** and **Br-BS** in 50 wt%-doped films at 77 K.

Table S4 Excitation energies for the S_1 and T_1 states and S_1–T_1 energy splittings

	**1**[d]	**2**[d]	**3**[d]	**4**[d]	**MPASi**[e]	**MFASi**[e]	**MPAGe**[e]	**MCz**[e]	**Br-BS**[e]
E_S[a] [eV]	2.85	2.82	2.92	2.87	-	-	-	-	-
E_T[b] [eV]	2.77	2.76	2.81	2.76	3.01	2.78	3.07	3.04	2.92 (2.78)[d]
ΔE_{ST}[c] [eV]	0.08	0.06	0.11	0.11	-	-	-	-	-

[a]The lowest singlet excitation energy estimated from the onset energy of fluorescence spectrum at 300 K. [b]The lowest triplet excitation energy estimated from the onset energy of phosphorescence spectrum at 77 K. [c]$\Delta E_{ST} = E_S - E_T$. [d]Measured in the 50 wt%-doped films in PPF. [e]Measured in the frozen toluene solution.

5. Computational Methods

Geometry optimization for **1–5** in the S_0 state were performed using the B3LYP functional with the 6-31G(d) basis set in the gas phase, implemented in the Gaussian 16 program package.[6] The transition-state (TS) geometries were optimized using TS keyword. It was confirmed that the transition states show only one negative imaginary frequency and the optimized structures displaced in the direction of negative frequency from the TS geometries are identical to the QA or QE geometries. The population ratios between QE and QA were estimated by Boltzmann distribution as follows,

$$[QA](\%) = \frac{2 \times \exp\left(-\frac{\Delta G_{QA}}{k_b T}\right)}{2 \times \exp\left(-\frac{\Delta G_{QA}}{k_b T}\right) + \exp\left(-\frac{\Delta G_{QE1}}{k_b T}\right) + \exp\left(-\frac{\Delta G_{QE2}}{k_b T}\right)}$$

$$[QE](\%) = \frac{\exp\left(-\frac{\Delta G_{QE1}}{k_b T}\right) + \exp\left(-\frac{\Delta G_{QE2}}{k_b T}\right)}{2 \times \exp\left(-\frac{\Delta G_{QA}}{k_b T}\right) + \exp\left(-\frac{\Delta G_{QE1}}{k_b T}\right) + \exp\left(-\frac{\Delta G_{QE2}}{k_b T}\right)}$$

where ΔG are the relative Gibbs free energy changes, k_b is the Boltzmann constant, and T is the temperature (298 K). The population ratios were calculated considering that there are two enantiomeric isomers in QA for the rotation of donor and acceptor units.

The geometries of the S_1 and T_1 states were optimized using the TD-DFT method at the B3LYP/6-31G(d) level in the gas phase. The singlet and triplet excitation energies were calculated by TD-DFT calculations using the optimally tuned range-separated functional (LC-ωPBE) with the 6-31+G(d) basis set. The optimal range-separation parameter ω was obtained by minimizing the following equation,[7]

$$J^2 = \sum_{i=0}^{1} [\varepsilon_{HOMO}(N+i) + IP(N+i)]^2$$

where ε_{HOMO} is the energy of the HOMO and IP is the ionization potential for the N-electron system. For the optimization of the ω value, the single-point calculations based on the S_0 geometries optimized at B3LYP/6-31G(d) level were carried out for QE and QA of **1**. The optimal ω values of 0.15 and 0.14 were used in the TD-DFT LC-ωPBE calculations for QE of **1–3** and **4**, and QA of **1–3**, respectively.

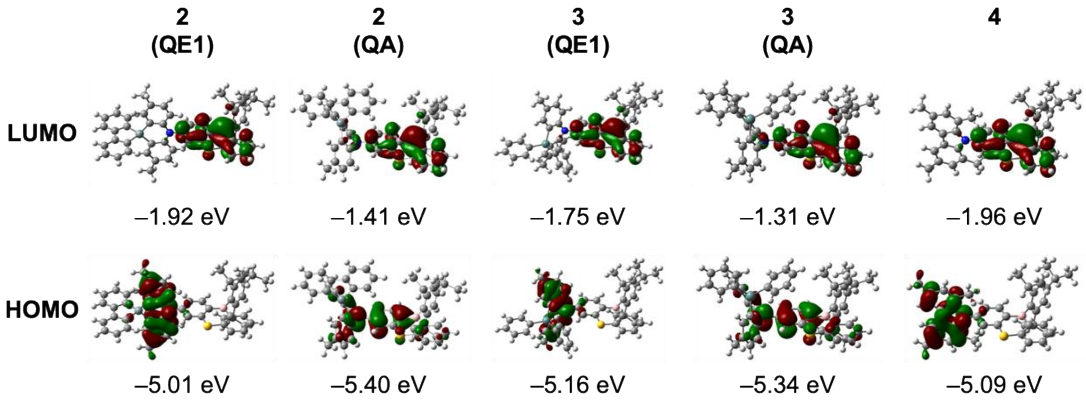

Fig. S15 Kohn–Sham molecular orbitals of **2–4** in the S_0 states and their energy levels, calculated at the B3LYP/6-31G(d) level.

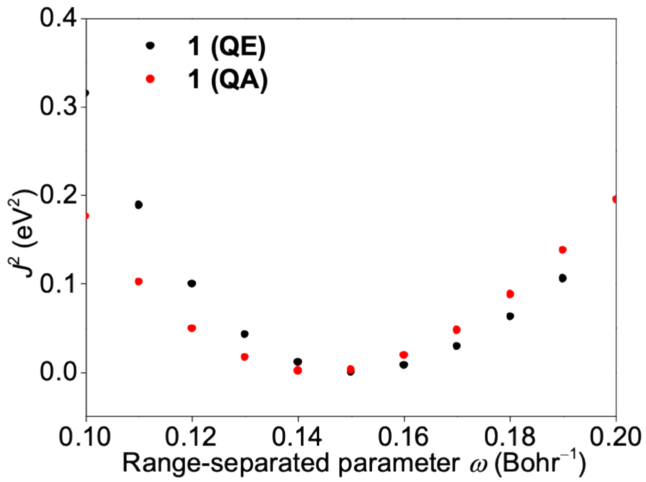

Fig. S16 J^2 values as a function of the range-separation parameter ω for QE and QA conformers of **1** calculated at the LC-ωPBE/6-31+G(d) level.

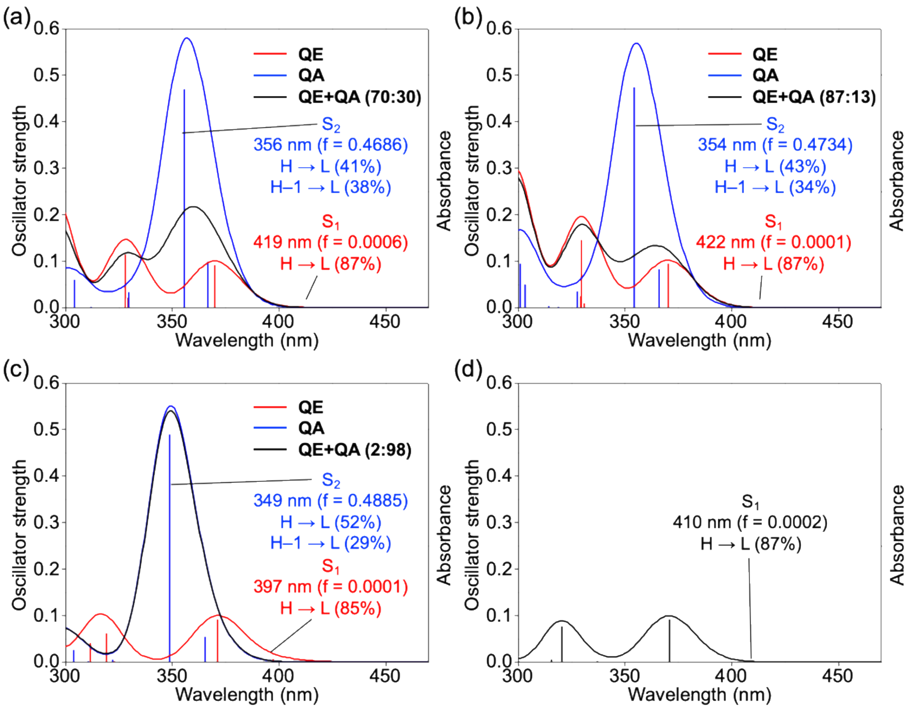

Fig. S17 The singlet excitation wavelengths and oscillator strengths (*f*), and absorption spectra calculated at the LC-*ω*PBE/6-31+G(d) level in the S_0 optimized geometries. H−*n* → L+*m* represents the HOMO−*n* to LUMO+*m* transitions. The values in parentheses imply the weights of transition configurations.

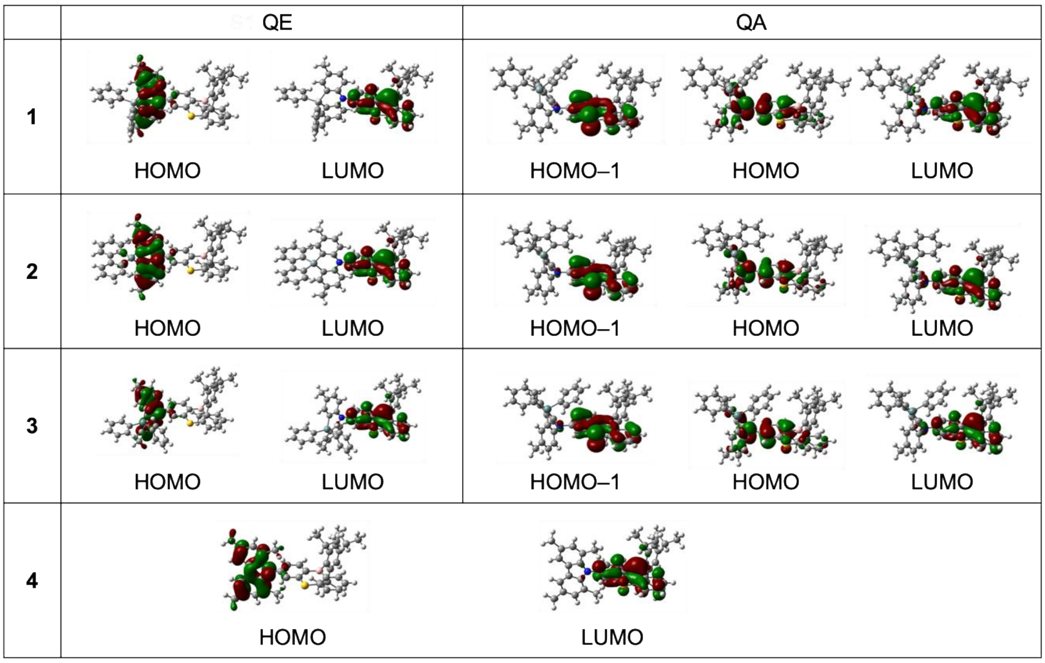

Fig. S18 Kohn–Sham molecular orbitals of **1–4** most involved in the vertical excitations for the S_1 states, calculated at the LC-*ω*PBE/6-31+G(d) level in the optimized S_0 geometries.

Table S5 TD-DFT calculation results in the optimized S_1 and T_1 geometries

	state	E [eV]	λ [eV]	E_S, E_T [eV]	f	main configuration	[%]	ΔE_{S1-T1} [meV]	ΔE_{S1-T1} [meV]
**1**	S_1	2.640	0.147	2.786	0.0001	H → L	89	37	26
	T_1	2.604	0.145	2.749	0.0000	H−1 → L<br>H → L	79<br>16		
	T_2	2.630	0.145	2.775	0.0000	H → L<br>H−1 → L	75<br>17		
**2**	S_1	2.665	0.153	2.818	0.0001	H → L	89	61	49
	T_1	2.605	0.152	2.757	0.0000	H−2 → L	90		
	T_2	2.654	0.152	2.806	0.0000	H → L	84		
**3**	S_1	2.619	0.120	2.739	0.0001	H → L	89	146	511
	T_1	2.498	0.095	2.593	0.0000	H–1 → L	97		
	T_2	3.010	0.095	3.104	0.0000	H → L	78		
**4**	S_1	2.729	0.137	2.866	0.0000	H → L	89	112	99
	T_1	2.617	0.137	2.754	0.0000	H−2 → L	93		
	T_2	2.716	0.137	2.853	0.0000	H → L	88		

The excitation energies (E), relaxation energies (λ), the energy levels of the excited states (E_S and E_T), f, transition configurations, and adiabatic ΔE_{ST} of **1–4** calculated at the LC-ωPBE/6-31+G(d) level in the S_1 and T_1 optimized geometries. E_S (E_T) = $E + \lambda$.

H−n → L+m represents the HOMO−n to LUMO+m transition.

6. OLED Fabrication and Evaluation

Indium tin oxide-coated glass substrates were cleaned with detergent, deionized water, acetone, and isopropanol. They were then treated with UV–ozone treatment for 30 min, before being loaded into a vacuum evaporation system. The organic layers were thermally evaporated on the substrates under vacuum ($< 1 \times 10^{-4}$ Pa) with an evaporation rate of < 1 Å s^{-1}. All of the layers were deposited through a shadow mask. The layer thickness and deposition rate were monitored *in situ* during deposition by an oscillating quartz thickness monitor. OLED properties were measured using a Keithley source meter 2400 and a Konica Minolita CS-2000. Luminance (L), external EL quantum efficiency (η_{ext}), current efficiency (η_c), and power efficiency (η_p) were corrected by Lambertian factors of the devices estimated from the angular dependence of the EL intensity.

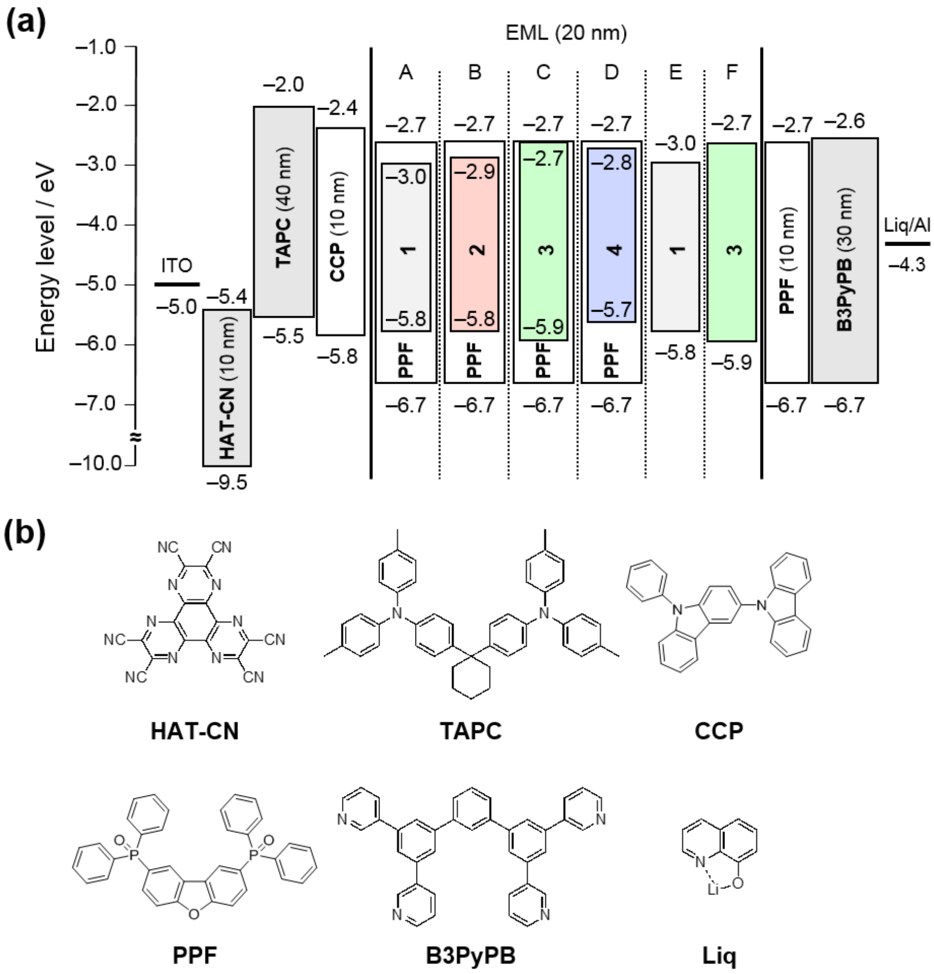

Fig. S19 (a) Schematic energy-level diagram for OLEDs based on **1–4** as emitters and (b) molecular structures of the materials used in the devices.

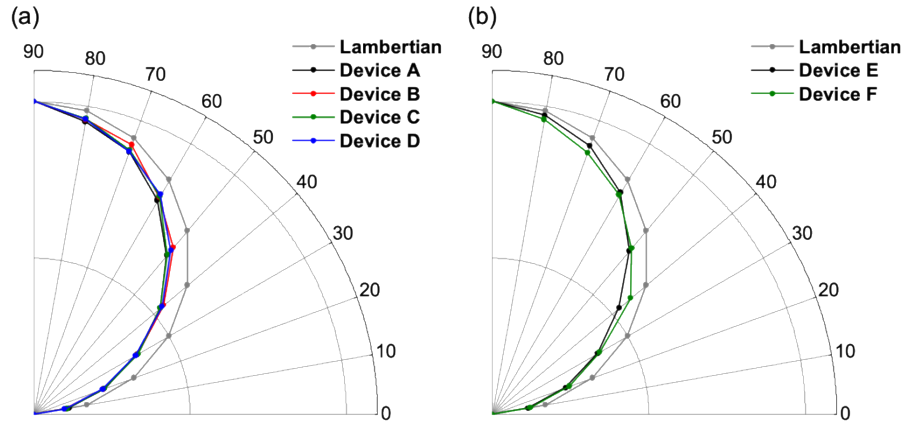

Fig. S20 The angular dependence of the EL intensities with the Lambertian distribution of (a) devices A–D and (b) E and F. Lambertian factors were calculated to be 0.835, 0.838, 0.847, 0.837, 0.854, and 0.873 for devices A–F, respectively.

7. References

1. F. S. Wekesa, N. Phadke, C. Jahier, D. B. Cordes and M. Findlater, *Synthesis*, 2014, **46**, 1046.
2. I. S. Park, K. Matsuo, N. Aizawa and T. Yasuda, *Adv. Funct. Mater.*, 2018, **28**, 1802031.
3. S. J. Aspin, S. Taillemaud, P. Cyr and A. B. Charette, *Angew. Chem. Int. Ed.*, 2016, **55**, 13833.
4. G. Sheldrick, *Acta Cryst., Sec. C*, 2015, **71**, 3.
5. I. S. Park, H. Komiyama and T. Yasuda, *Chem. Sci.*, 2017, **8**, 953.
6. Gaussian 16 (Revision A.03) M. J. Frisch, G. W. Trucks, H. B. Schlegel, G. E. Scuseria, M. A. Robb, J. R. Cheeseman, G. Scalmani, V. Barone, G. A. Petersson, H. Nakatsuji, X. Li, M. Caricato, A. V. Marenich, J. Bloino, B. G. Janesko, R. Gomperts, B. Mennucci, H. P. Hratchian, J. V. Ortiz, A. F. Izmaylov, J. L. Sonnenberg, D. Williams-Young, F. Ding, F. Lipparini, F. Egidi, J. Goings, B. Peng, A. Petrone, T. Henderson, D. Ranasinghe, V. G. Zakrzewski, J. Gao, N. Rega, G. Zheng, W. Liang, M. Hada, M. Ehara, K. Toyota, R. Fukuda, J. Hasegawa, M. Ishida, T. Nakajima, Y. Honda, O. Kitao, H. Nakai, T. Vreven, K. Throssell, J. A. Montgomery, Jr., J. E. Peralta, F. Ogliaro, M. J. Bearpark, J. J. Heyd, E. N. Brothers, K. N. Kudin, V. N. Staroverov, T. A. Keith, R. Kobayashi, J. Normand, K. Raghavachari, A. P. Rendell, J. C. Burant, S. S. Iyengar, J. Tomasi, M. Cossi, J. M. Millam, M. Klene, C. Adamo, R. Cammi, J. W. Ochterski, R. L. Martin, K. Morokuma, O. Farkas, J. B. Foresman and D. J. Fox, Gaussian, Inc., Wallingford CT, 2016.
7. H. Sun, C. Zhong and J.-L. Brédas, *J. Chem. Theory Comput.*, 2015, **11**, 3851.
